# *NTRK* fusion positive colorectal cancer is a unique subset of CRC with high TMB and microsatellite instability

**DOI:** 10.1002/cam4.4561

**Published:** 2022-05-04

**Authors:** Hui Wang, Zhi‐Wei Li, Qiuxiang Ou, Xue Wu, Misako Nagasaka, Yang Shao, Sai‐Hong Ignatius Ou, Yu Yang

**Affiliations:** ^1^ Department of Medical Oncology, Beijing Hospital, National Center of Gerontology, Institute of Geriatric Medicine Chinese Academy of Medical Sciences Beijing China; ^2^ Department of Internal Medicine Harbin Medical University Cancer Hospital Harbin China; ^3^ Geneseeq Research Institute Nanjing Geneseeq Technology Inc. Nanjing Jiangsu China; ^4^ Karmanos Cancer Institute Wayne State University Detroit Michigan USA; ^5^ School of Public Health Nanjing Medical University Nanjing Jiangsu China; ^6^ Chao Family Comprehensive Cancer Center University of California Irvine School of Medicine Orange California USA; ^7^ Department of Oncology, the Second Affiliated Hospital of Harbin Medical University Harbin Medical University Harbin China

**Keywords:** colorectal cancer, gene fusions, microsatellite instability, NTRK, *POLE/POLD1*, tumor mutation burden

## Abstract

TRK fusions are rare but targetable mutations which occur across a wide variety of cancer types. We report the prevalence of approximately 0.7% for *NTRK*‐positive colorectal cancer (CRC) by genetically profiling 2519 colonic and rectal tumors. The aberrations of *APC* and *TP53* frequently co‐occurred with *NTRK* gene fusions, whereas *RAS/BRAF* oncogenic alterations and *NTRK* fusions were almost always mutually exclusive. *NTRK*‐driven colorectal cancer patients demonstrated increased TMB (median = 53 mut/MB, 95% CI: 36.8–68.0 mut/MB), high microsatellite instability, and an enrichment for *POLE/POLD1* mutations when compared to molecularly unstratified colorectal cancer population. These data shed light on possible future approach of multimodality treatment regimen including TRK‐targeted therapy and immune checkpoint inhibitor therapy in *NTRK*‐positive CRCs.

## Graphical abstract

NTRK fusions positive colorectal cancer (CRC) are rare. NTRK‐positive CRC tumors demonstrated very high tumor mutation burden (median 53 mut/MB), microsatellite instability‐high (MSI‐H, 76%), and an enrichment of concurrent POLE and POLD1 mutations. These data may be informative in guiding molecularly driven treatment including targeted therapy and immunotherapy for treating NTRK+ CRC patients. Patients with MSI‐H or high TMB CRC should also be screened for NTRK fusions.

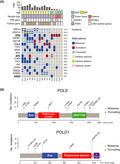

**What's New?**

*NTRK* fusions positive colorectal cancer (CRC) are rare (<1%). *NTRK*‐positive CRC tumors demonstrated very high tumor mutation burden (median 53 mut/MB), microsatellite instability‐high (MSI‐H, 76%), and an enrichment of concurrent *POLE* and *POLD1* mutations. These data may be informative in guiding molecularly driven treatment including targeted therapy and immunotherapy for treating *NTRK+* CRC patients. Patients with MSI‐H or high TMB CRC should also be screened for *NTRK* fusions.

## 1 INTRODUCTION

The *NTRK* genes (*NTRK1/2/3*) encode tropomyosin receptor kinase (trk) proteins (TrkA/B/C) which are mainly involved in neural development and homeostasis.^1^ TRK fusions are rare but targetable mutations which occur in both adults and children.^2^ Studies have shown that TRK inhibitors were able to produce durable responses in TRK fusion–positive cancer patients.^3^, ^4^ Currently, two first generation (1G) NTRK TKIs (larotrectinib,^5^, ^6^ entrectinib^7^) have been approved by the US Food and Drug Administration (FDA) for the treatment of both adult and pediatric cancers in a tumor‐agnostic manner. Furthermore, a number of next‐generation NTRK TKIs (selitrectinib [LOXO‐195],^8^ repotrectinib,^9^ and taletrectinib^10^) that can overcome acquired on‐target *NTRK* resistance mutations especially solvent‐front mutation to first‐generation NTRK TKIs^3^ are in clinical development.

There were previous reports that *NTRK+* colorectal cancer may represent a unique subset of CRC with high tumor mutation burden (TMB) and are more likely to be microsatellite unstable.^11^, ^12^ In this study, we analyzed the clinicopathologic and molecular characteristics of a large cohort of Chinese CRC patients through comprehensive genomic profiling using next‐generation sequencing from either tumor or blood samples, and identified the frequency, and clinicopathologic and genetic features, including tumor mutation burden (TMB) and microsatellite instability status (MSI), of *NTRK‐*driven colorectal cancers with the ultimate goal of further informing diagnostic and treatment decisions.

## 2 MATERIALS AND METHODS

### 2.1 Patients and samples

A series of 2519 consecutive colorectal cancer clinical cases were analyzed using comprehensive genomic profiling (CGP) in a Clinical Laboratory Improvement Amendments‐certified, College of American Pathologists accredited laboratory (422‐gene panel –GeneseeqOne™; 425‐gene panel – GeneseeqPrime™; Nanjing Geneseeq Technology, Jiangsu, China), as previously described.^13^ Detailed panel gene lists are provided in Table S1. While both panels could detect *NTRK1* fusions, GeneseeqPrime™ had the additional capacity of detecting *NTRK2/3* fusions, in which all exons (including flanking intronic regions) of *NTRK1/2/3* plus selected introns including *NTRK1* (introns 4, 7–13), *NTRK2* intron 12, and *NTRK3* introns 12–14 were covered. Furthermore, *ETV6* introns 4–6 were included for the detection of *ETV6‐NTRK3* fusions. We identified patients with *NTRK+* fusions by searching using natural language search tool in the Laboratory Information Management System (LIMS) database. Relevant demographic and clinical data were extracted from the database for these cases, including age, gender, date of diagnosis, histology type, pathological stage, and evaluation of treatment response per reports by clinical investigators.

For tumor tissue samples, the pathologic diagnosis and tumor content of each case was confirmed by pathologists. Peripheral blood of 8–10 ml was collected in EDTA‐coated tubes (BD Biosciences) and centrifuged at 1800 g for 10 min within 2 h of collection to separate the plasma for circulating tumor DNA (ctDNA) extraction and white blood cells for genomic DNA extraction as germline control. In accord with the Declaration of Helsinki, written informed consent was collected from each patient prior to sample collection. This study was approved by the ethics committee of the Second Affiliated Hospital of Harbin Medical University, Harbin, China.

### 2.2 DNA extraction and targeted enrichment

Genomic DNA from the white blood cells were extracted using the DNeasy Blood & Tissue Kit (Qiagen), while genomic DNA of fresh or *formalin‐fixed paraffin‐embedded* (*FFPE*) tumor specimens was purified using the QIAamp DNA FFPE Tissue Kit (Qiagen). All DNA was quantified using the dsDNA HS Assay Kit on a Qubit Fluorometer (Life Technologies). Sequencing libraries were prepared using the KAPA Hyper Prep Kit (Roche), as described previously.^10^ Indexed DNA libraries were pooled together for probe‐based hybridization capture of the targeted gene regions covered by different gene panels.

### 2.3 Sequencing data processing

Sequencing was performed on the Illumina HiSeq4000 platform (150 bp paired end sequencing) followed by data analysis as previously described.^13^, ^14^ The sequencing coverage and quality statistics of patients' tumor or plasma specimens are summarized in Table S2. The corresponding whole blood control samples were sequenced to a median depth of 240X (range: 177X–384X). Specifically, sequencing data were analyzed by Trimmomatic^15^ to remove low‐quality (quality <15) or *N* bases, and then mapped to the human reference genome hg19 using the Burrows‐Wheeler Aligner (https://github.com/lh3/bwa/tree/master/bwakit). PCR duplicates were removed by Picard (available at: https://broadinstitute.github.io/picard/). The Genome Analysis Toolkit (GATK) (https://software.broadinstitute.org/gatk/) was used to perform local realignments around indels and base quality reassurance. SNPs and indels were analyzed by VarScan2^16^ and HaplotypeCaller/UnifiedGenotyper in GATK, with the mutant allele frequency (MAF) cutoff as 0.5% for tumor tissue/FFPE samples, 0.1% for plasma cfDNA samples, and a minimum of three unique mutant reads. Common SNPs were excluded if they were present in >1% population frequency in the 1000 Genomes Project or the Exome Aggregation Consortium (ExAC) 65,000 exomes database. The resulting mutation list was further filtered by an in‐house list of recurrent artifacts based on a normal pool of whole blood samples. Gene fusions were identified by FACTERA.^17^

Tumor mutation burden (TMB) was calculated based on the number of non‐synonymous somatic mutations in the coding region per megabase.^13^ Microsatellite (MS) status of tumor sample was determined on the overall stability of MS loci tested in the panel. A sample was reported as microsatellite instable (“MSI”) if ≥40% of the MS loci display instability, or as “MSS” if <40% of the MS loci display instability.

### 2.4 PD‐L1 staining

PD‐L1 staining was performed using the monoclonal mouse antihuman PD‐L1 antibody (clone 22C3, Cat No. M3653; Dako). A minimum of 100 viable tumor cells must be present in the specimen slide for the PD‐L1 expression to be calculated with complete or partial membrane staining. PD‐L1 assay results were interpreted according to the scoring guidelines as previously described.^18^

## 3 RESULTS

### 3.1 Incidence of *NTRK* ‐positive colorectal cancer and fusion partners

**FIGURE 1:**
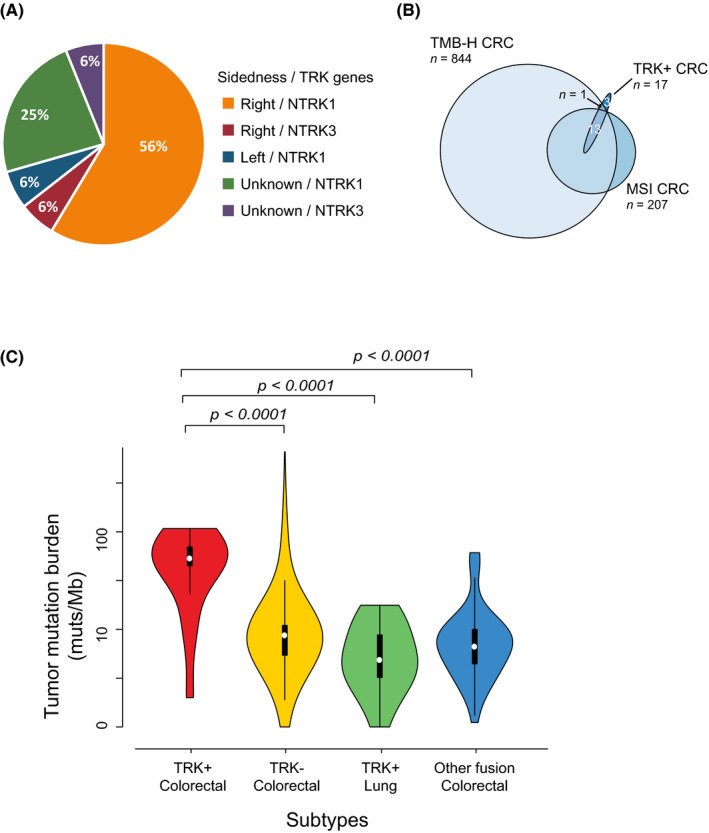
*NTRK* fusions in colorectal cancer. (A). Colon tumor site. (B). Venn diagram of the relationships between *NTRK+* colorectal cancer (CRC), high tumor mutational burden (TMB), and positive microsatellite instability status (MSI). (C). The comparison of TMB between *NTRK+* CRC, molecularly unstratified CRC, *NTRK+* non‐CRC, and CRC that carried other kinase fusions

**FIGURE 2:**
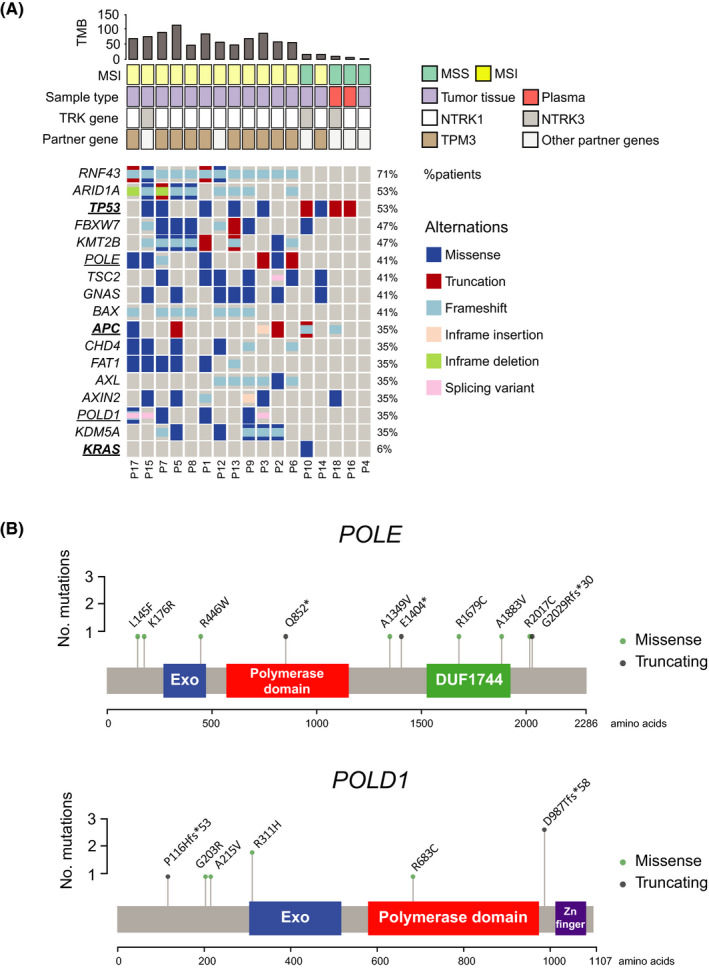
Genomic features observed in *NTRK+* colorectal cancers. (A) Co‐mutation plot illustrating alterations with the occurrence of at least one third of the *NTRK+* cohort. Each column represents a *NTRK‐*fusion positive patient. Alteration types are color‐coded shown on the right panel. Patient's clinicopathological features and tumor mutation burden were shown on top of the co‐mutation plot. (B) The lollipop plot mapping identified mutations of *POLD1* or *POLE* to protein sequences

From April 2016 to May 2020, a total of 2940 unique clinical colorectal cancer fresh or FFPE tumor samples derived from 2519 patients were successfully evaluated with comprehensive genomic profiling using next‐generation sequencing. Among them, a total of 17 *NTRK+* colorectal cancer patients were identified, including 14 cases of *NTRK1+* CRCs and three cases of *NTRK3+* CRCs (Figure 1A). The overall incidence of *NTRK+* fusion positive CRC was thus approximately 0.7% (17/2519). The characteristics of the patients are summarized in Table 1, and a detailed description of each patient's demographic and clinical information are provided in Table 2. The median age of diagnosis was 65 years (range: 38–76 years, Table 1). The cohort had 16 cases of colon cancer and one case of rectal cancer, and more than half (58.8%) were confirmed of right‐sided tumors (ascending colon) (Figure 1A). As provided in Table 2, *TPM3* was the most common fusion partner (11/14) of *NTRK1*, and the other detected partners included *LMNA* (*n* = 2) and *TRP* (*n* = 1). *NTRK1* rearrangements most frequently occurred in *NTRK1* introns 7, 8, 9, 10, and 11. *NTRK3+* fusions accounted for the remaining three *NTRK+* CRC, in all cases that *NTRK3* (exon 14) was fused to *ETV6*, *RUNX1* (Figure S1A), and *CSNK1G1* (Figure S1B), respectively. Neither *RUNX1‐NTRK3* nor *CSNK1G1‐NTRK3* fusions were previously reported in CRC or any other cancer types. The patient P10, who was detected of *RUNX1‐NTRK3* (MAF: 8.9%, also carried a *KRAS* Q61R point mutation (Figure 2A). The patient P18 harbored a novel *CSNK1G1‐NTRK3* fusion at a MAF of 2.7% with concurrent deleterious mutations of *TP53* and *APC* (Figure 2A), although no canonical driver mutations were identified.

**TABLE 1:** Patient overview

Characteristics	*TRK+* CRC (*N* = 17)	*NTRK1+* subset (*N* = 14)
Age of onset, median, years	65 (range: 38–76)	67 (range: 52–76)
Sex, *n* (%)		
Female	9 (52.9%)	8 (57.1%)
Male	8 (47.1%)	6 (42.9%)
Stage, *n* (%)		
III‐IV	5 (29.4%)	4 (28.6%)
n.d.	12 (70.6%)	10 (71.4%)
NTRK kinases, *n* (%)		
*NTRK1*	14 (82.3%)	14 (100%)
*NTRK3*	3 (17.7%)	—
Tumor site, *n* (%)		
Colon		
Right‐sided	11 (64.7%)	9 (71.5%)
n.d.	5 (29.4%)	4 (28.5%)
Rectum	1 (5.8%)	0
TMB, median, mut/MB	53 (range: 2–108)	53 (range: 2–108)
MSI‐positive, *n* (%)	13 (76.5%)	12 (85.7%)

Abbreviation: n.d., not determined.

**TABLE 2:** Clinical and demographic data of 17 *NTRK+* colorectal cancer patients

ID	Age	Sex	Stage	Site	Treatment history	TRK gene	Fusion partner	Fusion form	Breakpoint1	Breakpoint2	Allele frequency (P, plasma; F, FFPE)	Sample type	Molecular assay	TMB^a^ (mut/MB)	MSI status	PD‐L1 (TPS, CPS)
P1	74	M	NA	Colon‐transverse	NA	NTRK1	TPM3	TPM3:exon10‐NTRK1:exon9	1:154139441	1:156843913	12.20%	Tissue	422 gene panel	56	MSI	<1%, 2%
P2	67	F	IV	Colon‐ascending	Chemotherapy (Capecitabine, 4 mo, PD)	NTRK1	TPM3	NTRK1:exon8‐TPM3:exon7	1:156843713	1:154132660	16.50%	FFPE	422 gene panel	53	MSI	NA
								TPM3:exon6‐NTRK1:exon8	1:154132662	1:15684371512.30%						
P3	69	F	NA	Colon‐ascending	NA	NTRK1	TPM3	TPM3:exon6‐NTRK1:exon8	1:154130478	1:156843696	23.57%	Tissue	425 gene panel	75	MSI	2%, 7%
P4	53	M	NA	Colon‐site NA	Surgery	NTRK1	LMNA	LMNA:exon7‐NTRK1:exon11	1:156106224	1:156844785	32.27%	FFPE	425 gene panel	2	MSS	NA
P5	67	M	NA	Colon‐site NA	NA	NTRK1	TPM3	TPM3:exon6‐NTRK1:exon10	1:154134168	1:156844322	27.80%	FFPE	425 gene panel	108	MSI	NA
P6	67	F	NA	Colon‐site NA	NA	NTRK1	TPM3	TPM3:exon10‐NTRK1:exon8	1:154138026	1:156843608	30.30%	FFPE	425 gene panel	49	MSI	NA
P7	75	F	III	Colon‐ascending	Surgery	NTRK1	TPM3	TPM3:exon10‐NTRK1:exon9	1:154139680	1:156844015	9.53%	FFPE	425 gene panel	80	MSI	NA
P8	71	F	NA	Colon‐ascending	Surgery	NTRK1	TPM3	TPM3:exon6‐NTRK1:exon9	1:154134245	1:156843862	22.75%	FFPE	425 gene panel	45	MSI	NA
P9	63	M	IV	Colon‐ascending	Chemotherapy (CAPOX, 5 mo, PD), surgery	NTRK1	TPM3	NTRK1:exon8‐TPM3:exon7	1:156844130	1:154134285	26.44%	FFPE	425 gene panel	60	MSI	NA
P10	55	M	NA	Rectum	Chemotherapy (CAPOX, 6 mo, PD)	NTRK3	RUNX1	RUNX1:exon4‐NTRK3:exon14	21:36258226	15:88668341	8.90%	FFPE	425 gene panel	11	MSS	NA
P12	52	F	NA	Colon‐ascending	Surgery	NTRK1	LMNA	LMNA:exon8‐NTRK1:exon12	1:156106765	1:156844901	38.60%	FFPE	425 gene panel	51	MSI	NA
P13	76	F	NA	Colon‐site NA	NA	NTRK1	TPM3	TPM3:exon10‐NTRK1:exon12	1:154138749	1:156845155	3.4% (P), 9.7% (F)	FFPE & Plasma	425 gene panel	45 (F)	MSI	NA
								TPM3:exon10‐NTRK1:exon12	1:154138750	1:156845151	1.8% (P), 11% (F)					
P14	63	M	NA	Colon‐ascending	Surgery	NTRK1	TPM3	TPM3:exon10‐NTRK1:exon8	1:154134718	1:156843508	0.45% (P), 64.5% (F)	FFPE&Plasma	425 gene panel	12 (F)	MSI	NA
P15	57	F	NA	Colon‐ascending	Surgery	NTRK3	ETV6	ETV6:exon5‐NTRK3:exon14	12:12035081	15:88484921	27.80%	FFPE	425 gene panel	73	MSI	NA
									NTRK3:exon13‐ETV6:exon6	15:88484917	12:12035083	10.40%				
P16	65	M	III	Colon‐ascending	Chemotherapy (+bevacizumab, 3 yr, PD)	NTRK1	TRP	TPR:exon21‐NTRK1:exon10	1:186317772	1:156844344	23.94%	Plasma	425 gene panel	4 (P)	MSS	NA
								NTRK1:exon9‐TPR:exon22	1:156844343	1:186317771	3.04%					
P17	61	F	NA	Colon‐ascending	NA	NTRK1	TPM3	TPM3:exon10‐NTRK1:exon9	1:154134606	1:156843950	21.37%	FFPE	425 gene panel	67 (F)	MSI	25%, 30%
								TPM3:exon10‐NTRK1:exon9	1:154134606	1:156843952	1.13%	Plasma				
P18	38	M	IV	Colon‐site NA	NA	NTRK3	CSNK1G1	CSNK1G1:exon1‐NTRK3:exon14	15:64624388	15:88486523	5.00%	Plasma	425 gene panel	10 (P)	MSS	NA

Abbreviations: F, female; M, male; NA, not available; CAPOX, capecitabine and oxaliplatin; MSI, microsatellite instability; MSS, microsatellite stable; TPS, tumor proportion score; CPS, combined positive score.

^a^ Indicates that TMB was calculated based on the number of non‐synonymous mutations in the coding region per megabase.

Four patients (P2, P9, P10, and P16) received first‐line chemotherapy, six patients were treated with first‐line surgery, while the remaining seven cases were treatment‐naïve (Table 2). None of the patients received targeted therapy or immune checkpoint inhibitor therapy. All samples being analyzed by NGS were treatment‐naïve except in the cases of P2, P9, and P16 (Table 2).

### 3.2 Microsatellite instability status

Seventy‐six percent of the *NTRK+* CRC cohort was MSI‐positive tumors (microsatellite unstable) (Table 1), a rate much higher than that of the molecularly unstratified Chinese CRC population according to our database (8%, unpublished). Among the CRC samples that were microsatellite unstable (MSI), 6% was *NTRK* fusion positive (Figure 1B), in comparison to a rate 0.17% of *NTRK+* in the microsatellite stable (MSS) sub‐population. Furthermore, mutations of *POLE* or *POLD1* were detected in approximately 47% (8/17) of the *NTRK+* CRC subset and more than half of the patients (5/8) carried concurrent *POLE/POLD1* mutations including missense and truncating variants (Figure 2B). Of note, all *POLE+/POLD1+* tumors were microsatellite unstable.

### 3.3 DNA mismatch repair genes (MMR) status

Six patients were identified with somatic missense or frameshift aberrations of DNA mismatch repair (MMR) genes including *MLH1, MSH2, MSH6,* and *PMS2* (Table 2). Patient P1 and two additional patients (P6 and P12) also carried germline mutations of MMR genes (Table 2). All eight patients (47%, 8/17) who contained germline or somatic alterations of MMR genes were microsatellite unstable.

### 3.4 Tumor mutation burden (TMB)

*NTRK+* colorectal cancer patients had significantly higher tumor mutation burden (median 53 mutations per megabase[mut/MB], 95% CI: 36.8–68.0 mut/MB), Figure 1C) in comparison to that of the overall colorectal cancer population (median: 7.7 mut/MB, 95% CI: 11.8–14.2 mut/MB, *p* < 0.0001), *NTRK+* non‐CRC solid tumors (lung cancer) (median: 4 mut/MB, 95%CI: 2.4–7.7 mut/MB, *p* < 0.0001), or CRC samples harboring other oncogenic fusions including *ALK, ROS1*, and *FGFR* fusions (median: 6.6 mut/MB, 95%CI: 5.5–13 mut/MB, *p* < 0.0001, Figure 1C). All microsatellite unstable tumors had TMB of more than 10 mutations per megabase (TMB‐H) (Table 2). The patient P10 was MSS but had a TMB of 11 mutations per megabase (Table 2). Importantly, among all CRC samples that were TMB‐H (≥10 mut/MB), approximately 1.6% was *NTRK* fusion positive (Figure 1B).

### 3.5 PD‐L1 expression

In addition, we have also evaluated the PD‐L1 expression levels of three patients whose original samples were retrieved and remained adequate for testing (Figure S2). Both tumor proportion score (TPS) and combined positive score (CPS) were calculated (Table 2). All three patients were microsatellite unstable and had TMB of ≥10 mutations per megabase as well as CPS of 1 or higher, although the TPS appeared to be less than 1% in P1 (Table 2).

### 3.6 Genetic co‐alterations

*RNF43* was the most frequently mutated gene (71%) in *NTRK+* patients (Figure 2A), followed by *ARID1A* (53%), *TP53* (53%), and *KMT2B* (47%). The frequency of *TP53* (53%) or *APC* (35%) mutations, was relatively lower in the *NTRK+* cohort compared to that of the total CRC population (75% and 65%, respectively, unpublished). Notably, mutations of *RNF43* and *ARID1* were significantly enriched in *NTRK+* MSI‐positive tumors when compared to the *NTRK+* MSS counterparts (*p values* = 0.002 and 0.02, respectively, Fisher's exact test, Figure 2A). Mutated *APC* was identified in six out of 17 patients (35%) including missense, frameshift, in‐frame insertion, and truncations. Oncogenic *RAS/BRAF* aberrations were almost absent in the *NTRK+* CRC subset. The majority of *NTRK+* patients (15/17) were *RAS/BRAF wildtype*, except that a *KRAS* Q61R (mutant allele frequency [MAF]: 13.57%) was detected in P10 and a *BRAF* frameshift variant (A404Cfs*9, MAF: 22.65%) was identified in P9 (Table 2), although the clinical significance of the latter remained uncharacterized.

## 4 DISCUSSION

We demonstrated that CRC harboring *NTRK* fusion is rare with an approximate incidence of 0.7%. The *NTRK*‐positive cohort primarily consisted of *NTRK1* fusions. Three out of 17 *NTRK+* CRC were *NTRK3* fusions including two novel *NTRK3* fusions. No *NTRK2* fusions were identified. This is not due to insufficient “baiting” of *NTRK2* as probes to all kinase domain encoding exons of *NTRK2* as well as intron 12 were used and we have successfully identified *NTRK2* fusions from other tumor types in our database. Of note, while the aberrations of *APC* and *TP53* frequently co‐occurred with *NTRK* fusions, these fusions rarely coexisted with other activating driver mutations, consistent with what was previously reported for the *NTRK* rearrangement in a pan‐cancer setting by Rosen et al.^19^

The significance of our findings is that *NTRK+* CRC represents a unique molecular subtype of CRC with very high TMB (median 53 mut/MB, range 2–108 mut/MB) and were more likely to be microsatellite unstable. A total of eight patients (47% of the *NTRK+* CRC subset) harbored germline or somatic alterations of MMR genes. This dual molecular signature is not only unique to CRC, but also unique among other *NTRK+* solid tumors where the median TMB is 4 mut/MB for *NTRK+* lung cancer.

There is also important clinical implication of these dual molecular signature in *NTRK+* CRC is that there are two NTRK inhibitors (larotrectinib and entrectinib) approved in the US with several next‐generation TKIs being developed (selitrectinib, repotrectinib, and taletrectinib) to overcome the on‐target acquired resistance *NTRK* mutations in particularly the solvent‐front mutations. Additionally, the immune checkpoint inhibitor (ICI) pembrolizumab has now been approved for use first in a tumor‐agnostic manner in tumors that are microsatellite unstable or mismatch repair deficient that have progressed following prior treatment on May 23, 2017 and on June 29, 2020 approved for use as first‐line treatment of MSI‐high or MMR‐deficient CRC. Pembrolizumab was approved on June 27, 2020 in another tumor‐agnostic manner in tumors with high TMB (≥10 mut/MB). Thus, not only will most patients with this subset *NTRK+* CRC benefit from the current approved NTRK TKIs, but may also potentially benefit from ICIs. Notably, a prior study by Zou et al.^20^ reported that enriched CD8+ tumor‐infiltration T cells, quantified by using a DNA methylation‐based method, was associated with MSI‐H tumors in CRC cohorts and predicted better survival. However, it will require further investigation as to whether two molecular signatures (TMB and MSI) being positive, the response to pembrolizumab will be higher (additive or synergistic effect) than just having one molecular signature. Given the rarity of these *NTRK+* CRC, none of the 17 *NTRK+* colorectal cancer patients have been treated with pembrolizumab or any other ICIs.

At last, this study has a few limitations. First, we report an approximate frequency of 0.7% of *NTRK* fusions in colorectal cancer. Although this study was based on a large CRC population, it lacked a particular attention to potential accrual biases at different research sites owing to the study's real‐world and retrospective nature. Second, a more comprehensive diagnostic evaluation^21^ of the *NTRK* gene family is warranted. The current data can be supplemented by results of alternative diagnostic approaches, including targeted RNA testing,^22^ pan‐TRK immunohistochemical (IHC) staining,^23^ and DNA methylation analysis,^24^ which could particularly be useful in an scenario in which a novel rearrangement needs to be validated. In addition, a close follow‐up of patient's response to the following treatment is required, including TKI treatment and immunotherapy, if applicable.

## 5 CONCLUSIONS

*NTRK* fusions positive colorectal cancer are rare (0.7% of colorectal cancer). In addition to the absence of other known actionable driver mutations, *NTRK+* CRC tumors harbor very high tumor mutation burden (median 53 mut/MB), with most of them being microsatellite instability‐high (MSI‐H), and an enrichment of *POLE/POLD1* mutations. Of the 17 *NTRK+* colorectal cancer identified, 14 cases had *NTRK1*‐rearranged events with *TPM3* being the most frequent fusion partner, and the remaining three cases were *NTRK3+* fusion cases. These data may be informative in guiding molecularly driven treatment including targeted therapy and immunotherapy for treating *NTRK+* CRC patients. Patients with MSI‐H or high TMB CRC should also be screened for *NTRK* fusions.

## CONFLICT OF INTEREST

QO and XW are the employees of Nanjing Geneseeq Technology Inc., Nanjing, Jiangsu, China. MN received honorarium from Astra Zeneca and Tempus. YS is an employee and shareholder of Nanjing Geneseeq Technology Inc., Nanjing, Jiangsu, China. SHIO has received speaking/advisory honorarium from Pfizer, Merck, Roche/Genentech, Takeda/ARIAD, and AstraZeneca. SHIO is a stock owner and former member of the scientific advisory board of Turning Point Therapeutics, Inc. The remaining authors have no conflict of interest to declare.

## AUTHOR CONTRIBUTIONS

HW and ZL conceived and designed the study. QO analyzed the data. XW reviewed the data and revised the manuscript. YS provided the resources for the study. YY supervised the study. HW, ZL, and QO wrote the manuscript. MN and SHIO critically reviewed and revised the manuscript. All authors read and approved the final manuscript.

## ETHICS STATEMENT

In accord with the Declaration of Helsinki, written informed consent was collected from each patient prior to sample collection. This study was approved by the ethics committee of the Second Affiliated Hospital of Harbin Medical University, Harbin, China.

## Supporting information


Figure S1

Figure S2

Table S1

Table S2

Table S3

## Data Availability

Mutations identified in the 17 *NTRK+* colorectal cancer patients are provided in Table S3. Other data that supports the findings of this study are available from the corresponding author upon request.
