# Supplementary figures and images for: NTRK fusion positive colorectal cancer is a unique subset of CRC with high TMB and microsatellite instability
Source: Cancer Med. 2022 May 4;11(13):2541–9. doi: 10.1002/cam4.4561 (PMC9249987; doi:10.1002/cam4.4561)

Figure S1

(A) ***RUNX1*:exon 4-*NTRK3*: exon 14**

Chr 21: 36,258,206-36,258,246

Chr 15: 88,668,321-88,668,361

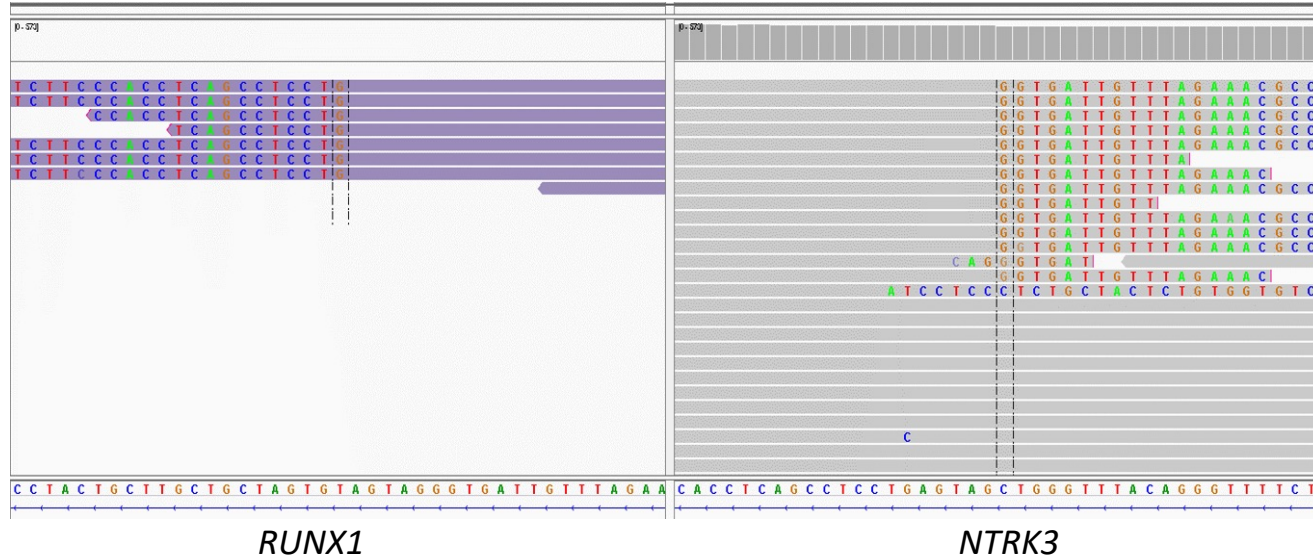

(B) ***CSNK1G1*: exon 1-*NTRK3*: exon 14**

Chr 15: 64,624,368-64,624,408

Chr 15: 88,486,503-88,486,543

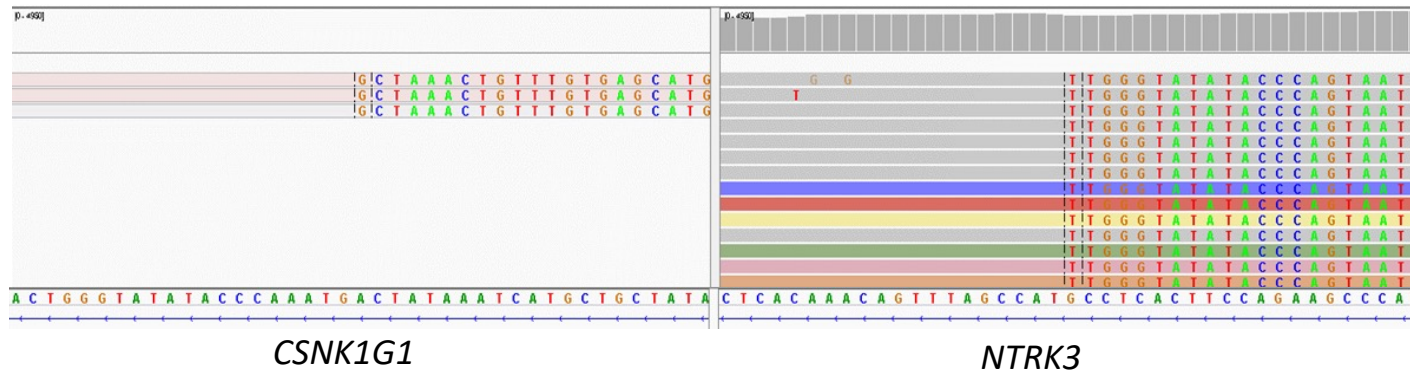

Supplement: Supplementary file 1 — Figure S1 [file CAM4-11-2541-s002.pdf]

Figure S2

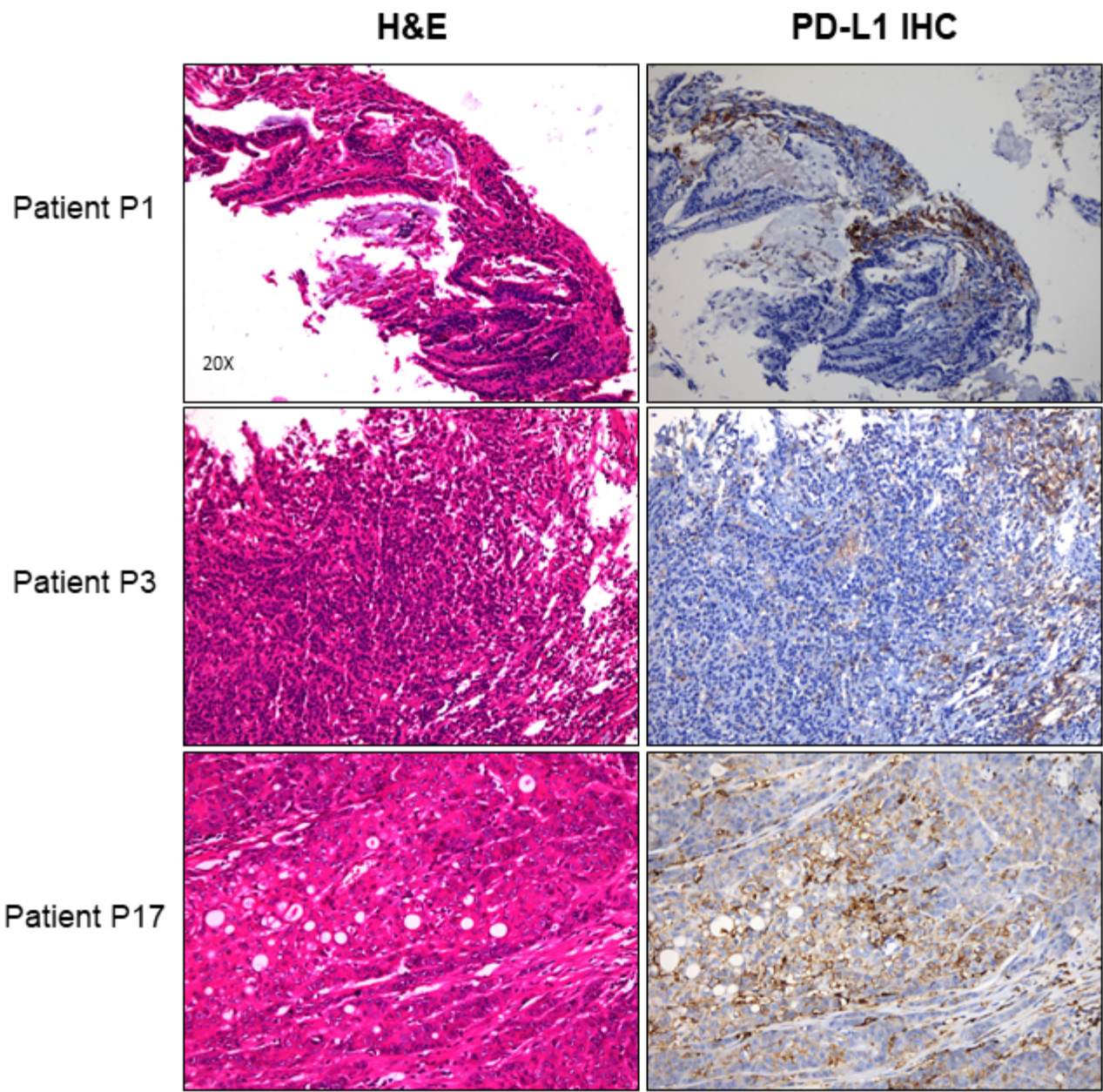

Supplement: Supplementary file 2 — Figure S2 [file CAM4-11-2541-s001.pdf]
