# Supplementary material for: NTRK fusion positive colorectal cancer is a unique subset of CRC with high TMB and microsatellite instability
Source: Cancer Med. 2022 May 4;11(13):2541–9. doi: 10.1002/cam4.4561 (PMC9249987; doi:10.1002/cam4.4561)
Supplement: Supplementary file 4 — Table S2 [file CAM4-11-2541-s003.pdf]

Supplementary Table S2. Sequencing statistics

Patient ID	Sample ID	Total number of sequenced reads	Total number of dedup reads	Percentage of on-target reads (%)	Median coverage (X) and range (per targeted base)	Percentage of targeted bases with coverage > 100X
P1	P1-T	6697740	4971063	77.49	471 (0-9577)	94.9
P2	P2-F	15091318	10403955	78.67	2111 (1-6747)	99.94
P3	P3-T	9711786	6537003	65.49	1011 (0-5243)	99.72
P4	P4-F	9448544	6283282	79.06	1155 (0-4160)	99.77
P5	P5-F	12356630	9229167	79.72	1511 (0-4730)	99.85
P6	P6-F	5317947	4357526	72.88	601 (0-1500)	99.45
P7	P7-F	8140642	6076175	78.95	1001 (0-2771)	99.73
P8	P8-F	6427672	4265403	76.52	591 (0-3123)	99.57
P14	P14-F	11359625	8310702	75.47	1131 (0-10399)	99.82
P15	P15-F	6263138	3842435	79.07	630 (0-6511)	99.45
P9	P9-F	16235458	9358118	79.65	1774 (0-19283)	99.79
P10	P10-F	8672148	5479063	72.47	860 (0-9924)	99.38
P12	P12-F	11885197	6545178	79.7	1332 (0-8710)	99.52
P13	P13-F	27181603	12973779	76.3	2895 (0-9177)	99.76
P14	P14-F	11359625	8310702	75.47	1131 (0-10399)	99.82
P15	P15-F	6263138	3842435	79.07	630 (0-6511)	99.45
P16	P16-PLA	38128383	16582034	86.15	5037 (0-26215)	99.89
P17	P17-F	2150564	1050120	86.95	204 (0-5989)	82.04
P18	P18-PLA	48573688	20750680	88.57	6041 (0-183897)	99.94
Note: Sample label, T, TISSUE; F, FFPE; P, PLASMA.						
