# Supplementary material for: NTRK fusion positive colorectal cancer is a unique subset of CRC with high TMB and microsatellite instability
Source: Cancer Med. 2022 May 4;11(13):2541–9. doi: 10.1002/cam4.4561 (PMC9249987; doi:10.1002/cam4.4561)
Supplement: Supplementary file 5 — Table S3 [file CAM4-11-2541-s004.pdf]

Supplementary Table S3. Mutations identified in 17 NTRK+ colorectal cancer patients as detected by a targeted gene panel containing over 400 cancer-relevant genes

Sample ID	Gene	Nucleotide change	Amino acid change	Chromosome start	Chromosome end	Mutation category	Allele frequency(%)
P1-T	PTEN	c.968delA	p.N323Mfs*21	chr10:89720812	chr10:89720812	frameshift	10.89
P1-T	FLT4	c.89delC	p.P30Rfs*3	chr5:180058748	chr5:180058748	frameshift	16.54
P1-T	BRCA1	c.1961delA	p.K654Sfs*47	chr17:41245587	chr17:41245587	frameshift	13.24
P1-T	SMO	c.2081delC	p.P694Lfs*82	chr7:128852004	chr7:128852004	frameshift	17.57
P1-T	AXIN2	c.1994delG	p.G665Afs*24	chr17:63532585	chr17:63532585	frameshift	18.17
P1-T	AURKA	c.987delT	p.F329Lfs*24	chr20:54945583	chr20:54945583	frameshift	15.74
P1-T	RNF43	c.1976delG	p.G659Vfs*41	chr17:56435161	chr17:56435161	frameshift	23.68
P1-T	PTEN	c.188delA	p.N63Tfs*36	chr10:89685289	chr10:89685289	frameshift	12.00
P1-T	BLM	c.1544delA	p.N515Mfs*16	chr15:91304139	chr15:91304139	frameshift	10.00
P1-T	AR	c.1832dupA	p.N611Kfs*12	chrX:66905910	chrX:66905911	frameshift	25.58
P1-T	DNMT3A	c.2255_2257delTCT	p.F752del	chr2:25463236	chr2:25463238	inframe deletion	21.98
P1-T	POLE	c.435G>T	p.L145F	chr12:133256226	chr12:133256226	missense	18.13
P1-T	FANCA	c.4301C>T	p.A1434V	chr16:89805076	chr16:89805076	missense	20.30
P1-T	LZTR1	c.1394C>T	p.A465V	chr22:21348253	chr22:21348253	missense	20.18
P1-T	BRCA2	c.2902G>T	p.G968C	chr13:32911394	chr13:32911394	missense	20.98
P1-T	ARID1B	c.6535G>A	p.V2179I	chr6:157528849	chr6:157528849	missense	17.06
P1-T	PLK1	c.892G>A	p.E298K	chr16:23695266	chr16:23695266	missense	15.15
P1-T	PRKCI	c.94T>G	p.Y32D	chr3:169940551	chr3:169940551	missense	2.20
P1-T	FAT1	c.5173C>A	p.L1725M	chr4:187542567	chr4:187542567	missense	15.87
P1-T	GRIN2A	c.136G>A	p.V46M	chr16:10274133	chr16:10274133	missense	21.21
P1-T	POLD1	c.2047C>T	p.R683C	chr19:50912816	chr19:50912816	missense	16.88
P1-T	ERBB4	c.3749G>A	p.R1250Q	chr2:212248518	chr2:212248518	missense	16.71
P1-T	SMARCA4	c.2108C>T	p.A703V	chr19:11118684	chr19:11118684	missense	25.05
P1-T	TP53	c.31G>A	p.E11K	chr17:7579882	chr17:7579882	missense	17.54
P1-T	ERBB4	c.2861T>A	p.V954D	chr2:212288885	chr2:212288885	missense	20.89
P1-T	PHOX2B	c.310A>G	p.T104A	chr4:41749485	chr4:41749485	missense	15.81
P1-T	TSC2	c.4390T>C	p.S1464P	chr16:2134613	chr16:2134613	missense	18.76
P1-T	FLT1	c.2276T>C	p.L759P	chr13:28919661	chr13:28919661	missense	15.25
P1-T	KMT2B	c.3572G>A	p.S1191N	chr19:36216164	chr19:36216164	missense	18.77
P1-T	MLH1	c.2156T>A	p.I719N	chr3:37092029	chr3:37092029	missense	16.42
P1-T	IGF1R	c.1423G>T	p.G475W	chr15:99452089	chr15:99452089	missense	18.65
P1-T	FGFR3	c.935C>T	p.A312V	chr4:1805423	chr4:1805423	missense	17.73
P1-T	GATA4	c.26C>T	p.A9V	chr8:11565847	chr8:11565847	missense	16.12
P1-T	FAT1	c.1637C>T	p.P546L	chr4:187629345	chr4:187629345	missense	2.20
P1-T	PRDM1	c.437A>G	p.H146R	chr6:106547200	chr6:106547200	missense	15.79
P1-T	ARID1B	c.5384G>A	p.R1795H	chr6:157527698	chr6:157527698	missense	20.97
P1-T	EXT2	c.1193C>T	p.A398V	chr11:44193180	chr11:44193180	missense	25.00
P1-T	NOTCH2	c.1063G>A	p.D355N	chr1:120512179	chr1:120512179	missense	18.94
P1-T	ERCC2	c.1486G>A	p.G496S	chr19:45858980	chr19:45858980	missense	18.85
P1-T	ERCC2	c.1847G>A	p.R616Q	chr19:45856059	chr19:45856059	missense	20.12
P1-T	PHOX2B	c.880G>A	p.V294I	chr4:41747889	chr4:41747889	missense	17.19
P1-T	HGF	c.1817T>A	p.I606N	chr7:81335010	chr7:81335010	missense	19.81
P1-T	EGFR	c.1878C>T	p.Y626=	chr7:55233128	chr7:55233128	splice region	21.16
P1-T	SETD2	c.1366C>T	p.R456*	chr3:47164760	chr3:47164760	nonsense	2.48
P1-T	CASP8	c.202C>T	p.R68*	chr2:202131411	chr2:202131411	nonsense	21.43
P1-T	RNF43	c.1111C>T	p.R371*	chr17:56436026	chr17:56436026	nonsense	16.61
P1-T	KMT2B	c.1008G>A	p.W336*	chr19:36211257	chr19:36211257	nonsense	18.85
P10-F	TP53	c.916C>T	p.R306*	chr17:7577022	chr17:7577022	nonsense	20.07

P10-F	SMAD4	c.1081C>T	p.R361C	chr18:48591918	chr18:48591918	missense	17.22
P10-F	KRAS	c.182A>G	p.Q61R	chr12:25380276	chr12:25380276	missense	13.57
P10-F	FBXW7	c.1394G>A	p.R465H	chr4:153249384	chr4:153249384	missense	12.18
P10-F	APC	c.3709C>T	p.Q1237*	chr5:112175000	chr5:112175000	nonsense	12.01
P10-F	PARK2	c.988G>A	p.V330M	chr6:161969981	chr6:161969981	missense	7.72
P10-F	EPHA3	c.2656G>A	p.G886S	chr3:89499486	chr3:89499486	missense	7.71
P10-F	APC	c.4188dupT	p.E1397*	chr5:112175475	chr5:112175476	frameshift	6.17
P10-F	ATR	c.2320dupA	p.I774Nfs*3	chr3:142274739	chr3:142274740	frameshift	5.73
P10-F	ABCB1	c.2903T>A	p.L968H	chr7:87148666	chr7:87148666	missense	1.14
P12-F	WISP3	c.678delA	p.K226Nfs*24	chr6:112389434	chr6:112389434	frameshift	28.15
P12-F	BAX	c.121delG	p.E41Rfs*19	chr19:49458971	chr19:49458971	frameshift	29.10
P12-F	RNF43	c.1976delG	p.G659Vfs*41	chr17:56435161	chr17:56435161	frameshift	29.79
P12-F	WISP3	c.677_678delAA	p.K226Mfs*21	chr6:112389434	chr6:112389435	frameshift	37.83
P12-F	ERBB3	c.3238delC	p.R1080Vfs*22	chr12:56494877	chr12:56494877	frameshift	12.88
P12-F	FANCM	c.4005delA	p.V1336Lfs*2	chr14:45645955	chr14:45645955	frameshift	33.48
P12-F	EPHA5	c.300dupA	p.V101Sfs*11	chr4:66467968	chr4:66467969	frameshift	30.28
P12-F	ARID1A	c.4217_4221delCCCCA	p.P1406Rfs*37	chr1:27100935	chr1:27100939	frameshift	32.94
P12-F	MLH3	c.2021delA	p.N674Ifs*6	chr14:75514338	chr14:75514338	frameshift	37.88
P12-F	TGFBR2	c.382_383delAA	p.K128Afs*3	chr3:30691872	chr3:30691873	frameshift	34.33
P12-F	QKI	c.401delA	p.K134Rfs*14	chr6:163899920	chr6:163899920	frameshift	10.81
P12-F	FBXW7	c.2001dupG	p.S668Efs*26	chr4:153244155	chr4:153244156	frameshift	25.49
P12-F	ERBB2IP	c.3163delG	p.E1055Kfs*15	chr5:65350306	chr5:65350306	frameshift	6.08
P12-F	KMT2A	c.2318delC	p.P773Rfs*8	chr11:118344186	chr11:118344186	frameshift	31.68
P12-F	TGFBR2	c.383dupA	p.P129Afs*3	chr3:30691871	chr3:30691872	frameshift	25.70
P12-F	AXL	c.195dupC	p.E66Rfs*25	chr19:41726644	chr19:41726645	frameshift	29.16
P12-F	NBN	c.689delT	p.L230*	chr8:90983414	chr8:90983414	frameshift	28.85
P12-F	BRIP1	c.917delA	p.N306Tfs*32	chr17:59885829	chr17:59885829	frameshift	34.24
P12-F	GATA4	c.366_368delCGC	p.A126del	chr8:11566176	chr8:11566178	inframe deletion	9.43
P12-F	MTOR	c.5145G>T	p.Q1715H	chr1:11194509	chr1:11194509	missense	30.38
P12-F	KMT2A	c.3479G>A	p.G1160D	chr11:118348826	chr11:118348826	missense	34.03
P12-F	FANCM	c.2521A>G	p.K841E	chr14:45644478	chr14:45644478	missense	33.85
P12-F	CHD4	c.5215G>A	p.A1739T	chr12:6687208	chr12:6687208	missense	33.02
P12-F	MED12	c.3970A>G	p.I1324V	chrX:70349987	chrX:70349987	missense	29.95
P12-F	SPRY4	c.488C>T	p.P163L	chr5:141694255	chr5:141694255	missense	28.13
P12-F	TEK	c.2377G>A	p.A793T	chr9:27206592	chr9:27206592	missense	34.51
P12-F	MTOR	c.2624A>G	p.E875G	chr1:11291382	chr1:11291382	missense	36.01
P12-F	PTCH1	c.4168G>T	p.G1390W	chr9:98209370	chr9:98209370	missense	34.11
P12-F	CYP2A6	c.1220A>G	p.N407S	chr19:41350619	chr19:41350619	missense	4.85
P12-F	TUBB3	c.1341G>T	p.Q447H	chr16:90002200	chr16:90002200	missense	32.55
P12-F	CBLB	c.2502A>T	p.E834D	chr3:105397342	chr3:105397342	missense	38.51
P12-F	KIT	c.2290C>A	p.L764I	chr4:55598093	chr4:55598093	missense	33.24
P12-F	GNAS	c.602G>A	p.R201H	chr20:57484421	chr20:57484421	missense	4.34
P12-F	CYP2D6	c.1001A>G	p.Q334R	chr22:42523468	chr22:42523468	missense	8.81
P12-F	NOTCH1	c.2647T>C	p.S883P	chr9:139405198	chr9:139405198	missense	29.07
P12-F	TSC2	c.445A>C	p.N149H	chr16:2104405	chr16:2104405	missense	4.37
P12-F	CCND1	c.536G>A	p.R179H	chr11:69458721	chr11:69458721	missense	28.83
P12-F	FGFR4	c.1046C>T	p.T349M	chr5:176519774	chr5:176519774	missense	36.71
P12-F	EXT1	c.1135G>A	p.V379I	chr8:118847712	chr8:118847712	missense	2.48
P12-F	PIK3CA	c.109C>A	p.L37I	chr3:178916722	chr3:178916722	missense	35.53
P12-F	KDM5A	c.1786C>T	p.R596C	chr12:438183	chr12:438183	missense	30.02

P12-F	PKHD1	c.10231G>T	p.V3411L	chr6:51524693	chr6:51524693	missense	34.39
P12-F	RNF43	c.849G>T	p.Q283H	chr17:56438144	chr17:56438144	missense	32.77
P12-F	SPOP	c.482T>C	p.V161A	chr17:47688818	chr17:47688818	missense	27.20
P12-F	FGFR4	c.92-1G>A	-	chr5:176517390	chr5:176517390	splice acceptor	2.62
P12-F	STAG2	c.1417-1G>A	-	chrX:123195073	chrX:123195073	splice acceptor	43.15
P12-F	PRKCI	c.478C>T	p.R160*	chr3:169988236	chr3:169988236	nonsense	31.11
P13-F	PREX2	c.2078delG	p.G693Dfs*10	chr8:69000006	chr8:69000006	frameshift	9.68
P13-F	FAT1	c.8635_8636delAA	p.K2879Efs*7	chr4:187539104	chr4:187539105	frameshift	10.07
P13-F	RNF43	c.1932dupA	p.S645Ifs*102	chr17:56435204	chr17:56435205	frameshift	9.34
P13-F	ARID1A	c.5548dupG	p.D1850Gfs*4	chr1:27105930	chr1:27105931	frameshift	6.92
P13-F	ROS1	c.1087dupT	p.Y363Lfs*25	chr6:117715401	chr6:117715402	frameshift	9.08
P13-F	PDCD1	c.105dupC	p.T36Hfs*70	chr2:242795103	chr2:242795104	frameshift	8.67
P13-F	BAX	c.549dupC	p.A184Rfs*29	chr19:49464240	chr19:49464241	frameshift	8.42
P13-F	AXL	c.685delC	p.R229Vfs*21	chr19:41737102	chr19:41737102	frameshift	9.12
P13-F	RNF43	c.1976delG	p.G659Vfs*41	chr17:56435161	chr17:56435161	frameshift	8.00
P13-F	JAK3	c.2462_2463delAC	p.H821Pfs*21	chr19:17943626	chr19:17943627	frameshift	8.43
P13-F	KMT2B	c.3057delA	p.G1020Afs*4	chr19:36214398	chr19:36214398	frameshift	9.98
P13-F	ROS1	c.4150delA	p.T1384Pfs*5	chr6:117674324	chr6:117674324	frameshift	6.93
P13-F	SLC3A2	c.899delA	p.K300Rfs*31	chr11:62649529	chr11:62649529	frameshift	11.38
P13-F	PIK3C3	c.192dupT	p.A65Cfs*18	chr18:39537653	chr18:39537654	frameshift	9.41
P13-F	CDK12	c.1233_1235dupTGC	p.A413dup	chr17:37627306	chr17:37627307	inframe insertion	8.12
P13-F	MED12	c.3888C>G	p.C1296W	chrX:70349905	chrX:70349905	missense	9.69
P13-F	LRP1B	c.3163G>A	p.G1055R	chr2:141680690	chr2:141680690	missense	8.99
P13-F	ATM	c.7010G>A	p.C2337Y	chr11:108198406	chr11:108198406	missense	9.91
P13-F	FLT1	c.3179G>A	p.R1060Q	chr13:28893667	chr13:28893667	missense	7.62
P13-F	TERT	c.2911C>T	p.R971C	chr5:1260648	chr5:1260648	missense	9.76
P13-F	TNFSF11	c.61G>A	p.G21S	chr13:43148500	chr13:43148500	missense	8.76
P13-F	IGF1R	c.508G>A	p.V170M	chr15:99251204	chr15:99251204	missense	2.90
P13-F	JAK2	c.1425A>T	p.K475N	chr9:5069120	chr9:5069120	missense	9.03
P13-F	TP53	c.734G>A	p.G245D	chr17:7577547	chr17:7577547	missense	10.31
P13-F	SMAD2	c.190G>A	p.A64T	chr18:45422938	chr18:45422938	missense	8.57
P13-F	CDC73	c.13C>G	p.L5V	chr1:193091343	chr1:193091343	missense	10.69
P13-F	PIK3R2	c.250G>A	p.A84T	chr19:18266939	chr19:18266939	missense	10.91
P13-F	SMO	c.389A>G	p.Y130C	chr7:128843282	chr7:128843282	missense	8.02
P13-F	PIK3R2	c.2054T>A	p.L685Q	chr19:18279971	chr19:18279971	missense	8.15
P13-F	PIK3CA	c.3139C>T	p.H1047Y	chr3:178952084	chr3:178952084	missense	10.02
P13-F	MITF	c.307C>T	p.P103S	chr3:69928487	chr3:69928487	missense	7.99
P13-F	GNAS	c.940G>A	p.A314T	chr20:57429260	chr20:57429260	missense	8.10
P13-F	CHD8	c.4276G>A	p.D1426N	chr14:21869128	chr14:21869128	missense	9.34
P13-F	PREX2	c.3184C>T	p.R1062C	chr8:69028025	chr8:69028025	missense	6.70
P13-F	MDM4	c.1111G>A	p.V371I	chr1:204518448	chr1:204518448	missense	8.56
P13-F	RET	c.1942G>A	p.V648I	chr10:43609990	chr10:43609990	missense	7.49
P13-F	PLK1	c.1669C>T	p.R557W	chr16:23701241	chr16:23701241	missense	10.23
P13-F	CCNE1	c.953-1G>T	-	chr19:30313352	chr19:30313352	splice acceptor	8.16
P13-F	ESR1	c.1553+2T>C	-	chr6:152415705	chr6:152415705	splice donor	8.04
P13-F	KMT2B	c.3412C>T	p.R1138*	chr19:36215615	chr19:36215615	nonsense	9.19
P13-F	FBXW7	c.1577G>A	p.W526*	chr4:153247225	chr4:153247225	nonsense	9.07
P13-F	ARID2	c.1996C>T	p.Q666*	chr12:46243902	chr12:46243902	nonsense	8.08
P14-F	IGF1R	c.3526G>A	p.V1176M	chr15:99486220	chr15:99486220	missense	19.05
P14-F	GRM8	c.2057C>T	p.A686V	chr7:126173379	chr7:126173379	missense	11.31

P14-F	PARK2	c.320T>C	p.L107P	chr6:162683649	chr6:162683649	missense	4.34
P14-F	TOP2A	c.3420G>T	p.K1140N	chr17:38555058	chr17:38555058	missense	13.05
P14-F	TNFSF11	c.5G>A	p.R2H	chr13:43148444	chr13:43148444	missense	29.61
P14-F	GNAS	c.602G>A	p.R201H	chr20:57484421	chr20:57484421	missense	31.32
P14-F	TP53	c.542G>C	p.R181P	chr17:7578388	chr17:7578388	missense	48.38
P14-F	RASGEF1A	c.1360G>A	p.A454T	chr10:43691985	chr10:43691985	missense	39.68
P14-F	GRIN2A	c.3307C>A	p.R1103S	chr16:9858094	chr16:9858094	missense	17.84
P14-F	SMAD4	c.1082G>A	p.R361H	chr18:48591919	chr18:48591919	missense	27.63
P14-F	TSC2	c.2585C>T	p.A862V	chr16:2125839	chr16:2125839	missense	19.60
P15-F	PTEN	c.968delA	p.N323Mfs*21	chr10:89720812	chr10:89720812	frameshift	20.91
P15-F	KMT2B	c.649delC	p.R217Gfs*22	chr19:36210893	chr19:36210893	frameshift	21.22
P15-F	MEN1	c.207delC	p.D70Tfs*49	chr11:64577375	chr11:64577375	frameshift	21.72
P15-F	NSD1	c.4591dupA	p.M1531Nfs*4	chr5:176675268	chr5:176675269	frameshift	19.47
P15-F	TTF1	c.821delA	p.K274Sfs*149	chr9:135277388	chr9:135277388	frameshift	20.25
P15-F	CTCF	c.610delA	p.T204Qfs*18	chr16:67645339	chr16:67645339	frameshift	17.52
P15-F	ARID1A	c.3977dupC	p.Q1327Afs*11	chr1:27100175	chr1:27100176	frameshift	18.00
P15-F	BLM	c.3651delA	p.K1217Nfs*62	chr15:91347484	chr15:91347484	frameshift	15.24
P15-F	IGF2	c.518dupC	p.E174Rfs*50	chr11:2154241	chr11:2154242	frameshift	21.07
P15-F	RNF43	c.1976delG	p.G659Vfs*41	chr17:56435161	chr17:56435161	frameshift	43.42
P15-F	EZR	c.1008_1009dupGA	p.K337Rfs*5	chr6:159191876	chr6:159191877	frameshift	18.10
P15-F	ARID1A	c.3977delC	p.P1326Rfs*155	chr1:27100176	chr1:27100176	frameshift	22.14
P15-F	FBXW7	c.1417dupA	p.R473Kfs*4	chr4:153249360	chr4:153249361	frameshift	20.71
P15-F	MED12	c.6306_6308dupGCA	p.Q2115dup	chrX:70361097	chrX:70361098	inframe insertion	15.55
P15-F	MED12	c.3046C>T	p.R1016C	chrX:70347807	chrX:70347807	missense	27.66
P15-F	NBN	c.1492C>T	p.P498S	chr8:90965825	chr8:90965825	missense	22.75
P15-F	PGR	c.495G>T	p.M165I	chr11:100999307	chr11:100999307	missense	23.39
P15-F	FGFR3	c.676T>C	p.Y226H	chr4:1803407	chr4:1803407	missense	18.30
P15-F	ARID1A	c.1717C>T	p.L573F	chr1:27058009	chr1:27058009	missense	22.33
P15-F	EPHA5	c.2573C>T	p.A858V	chr4:66213857	chr4:66213857	missense	24.78
P15-F	PLCB4	c.2452A>G	p.I818V	chr20:9404563	chr20:9404563	missense	24.23
P15-F	YAP1	c.512T>C	p.V171A	chr11:101985065	chr11:101985065	missense	27.65
P15-F	CDA	c.305G>A	p.C102Y	chr1:20940373	chr1:20940373	missense	2.45
P15-F	PDGFRB	c.373G>A	p.V125M	chr5:149514571	chr5:149514571	missense	24.48
P15-F	TET2	c.4118C>T	p.A1373V	chr4:106190840	chr4:106190840	missense	21.55
P15-F	IRF2	c.112T>C	p.W38R	chr4:185340698	chr4:185340698	missense	2.97
P15-F	GRM8	c.1538C>T	p.A513V	chr7:126173898	chr7:126173898	missense	17.27
P15-F	AIP	c.614C>T	p.A205V	chr11:67257654	chr11:67257654	missense	19.03
P15-F	PTEN	c.287C>A	p.P96Q	chr10:89692803	chr10:89692803	missense	12.50
P15-F	BMPR1A	c.1036C>T	p.H346Y	chr10:88679096	chr10:88679096	missense	17.67
P15-F	SDC4	c.139G>A	p.D47N	chr20:43964482	chr20:43964482	missense	21.07
P15-F	PKHD1	c.1384G>T	p.A462S	chr6:51923249	chr6:51923249	missense	25.35
P15-F	FLT4	c.371C>T	p.T124M	chr5:180057584	chr5:180057584	missense	19.92
P15-F	FAT1	c.13442G>A	p.R4481Q	chr4:187510071	chr4:187510071	missense	24.28
P15-F	AXIN2	c.1685C>T	p.P562L	chr17:63533469	chr17:63533469	missense	23.00
P15-F	FGFR3	c.791C>T	p.T264M	chr4:1803613	chr4:1803613	missense	17.96
P15-F	RNF43	c.1445G>A	p.C482Y	chr17:56435692	chr17:56435692	missense	18.77
P15-F	EXT1	c.300G>T	p.M100I	chr8:119122986	chr8:119122986	missense	25.80
P15-F	TP53	c.842A>G	p.D281G	chr17:7577096	chr17:7577096	missense	2.28
P15-F	CHD4	c.3520T>G	p.F1174V	chr12:6697061	chr12:6697061	missense	22.34
P15-F	PTPN13	c.1499G>T	p.R500L	chr4:87643478	chr4:87643478	missense	22.22

P15-F	MYD88	c.860G>A	p.C287Y	chr3:38182707	chr3:38182707	missense	23.58
P15-F	POLE	c.1336C>T	p.R446W	chr12:133250184	chr12:133250184	missense	22.29
P15-F	STAG2	c.3027A>T	p.K1009N	chrX:123217373	chrX:123217373	missense	19.44
P15-F	HDAC9	c.1411G>A	p.E471K	chr7:18688265	chr7:18688265	missense	23.98
P15-F	ERCC2	c.2246C>T	p.T749M	chr19:45854924	chr19:45854924	missense	21.70
P15-F	DPYD	c.670G>A	p.G224S	chr1:98164917	chr1:98164917	missense	22.12
P15-F	SUFU	c.1274C>T	p.A425V	chr10:104377163	chr10:104377163	missense	22.89
P15-F	KIT	c.148G>A	p.V50M	chr4:55561758	chr4:55561758	missense	17.24
P15-F	FANCG	c.1802G>A	p.R601H	chr9:35074172	chr9:35074172	missense	20.72
P15-F	NTRK2	c.1831C>A	p.L611I	chr9:87563443	chr9:87563443	missense	20.74
P15-F	TP53	c.512A>G	p.E171G	chr17:7578418	chr17:7578418	missense	5.33
P15-F	FAT1	c.9002G>A	p.G3001D	chr4:187538232	chr4:187538232	missense	19.51
P15-F	HDAC9	c.1886C>T	p.A629V	chr7:18767231	chr7:18767231	missense	19.36
P15-F	BAI3	c.353T>C	p.F118S	chr6:69348920	chr6:69348920	missense	5.63
P15-F	IRF2	c.587C>T	p.P196L	chr4:185320176	chr4:185320176	missense	2.22
P15-F	LHCGR	c.1123A>G	p.M375V	chr2:48915813	chr2:48915813	missense	5.47
P15-F	GSTT1	c.319C>T	p.R107W	chr22:24379393	chr22:24379393	missense	19.80
P15-F	SETBP1	c.1147G>A	p.A383T	chr18:42530452	chr18:42530452	missense	2.56
P15-F	PALLD	c.1106C>A	p.S369Y	chr4:169602501	chr4:169602501	missense	21.00
P15-F	PRF1	c.146A>G	p.D49G	chr10:72360513	chr10:72360513	missense	25.59
P15-F	MED12	c.5164C>T	p.R1722W	chrX:70356269	chrX:70356269	missense	18.52
P15-F	LRP1B	c.10465A>G	p.I3489V	chr2:141143528	chr2:141143528	missense	18.00
P15-F	GNAS	c.1040G>T	p.R347M	chr20:57485739	chr20:57485739	missense	18.14
P15-F	POLD1	c.2959delG	p.D987Tfs*58	chr19:50919866	chr19:50919866	splice acceptor	24.93
P15-F	XPC	c.900+1G>A	-	chr3:14206312	chr3:14206312	splice donor	19.89
P15-F	CDKN2A	c.172C>T	p.R58*	chr9:21971186	chr9:21971186	nonsense	25.56
P16-PLA	SMAD4	c.1081C>T	p.R361C	chr18:48591918	chr18:48591918	missense	6.48
P16-PLA	TP53	c.708C>A	p.Y236*	chr17:7577573	chr17:7577573	nonsense	4.98
P16-PLA	AR	c.1417G>A	p.G473S	chrX:66766405	chrX:66766405	missense	2.79
P16-PLA	ATM	c.1977G>T	p.K659N	chr11:108124619	chr11:108124619	missense	2.47
P17-F	POLD1	c.342delG	p.P116Hfs*53	chr19:50905055	chr19:50905055	frameshift	14.84
P17-F	RUNX1T1	c.1101delA	p.G368Vfs*22	chr8:92998419	chr8:92998419	frameshift	14.29
P17-F	ATR	c.666delT	p.F222Lfs*11	chr3:142281578	chr3:142281578	frameshift	24.14
P17-F	EPHA2	c.1379delC	p.P460Rfs*33	chr1:16462199	chr1:16462199	frameshift	12.43
P17-F	MECOM	c.1839delA	p.G614Efs*30	chr3:168833257	chr3:168833257	frameshift	7.95
P17-F	ARID1A	c.5715delA	p.K1905Nfs*18	chr1:27106100	chr1:27106100	frameshift	5.80
P17-F	PDGFRA	c.2438delA	p.N813Ifs*20	chr4:55151647	chr4:55151647	frameshift	8.51
P17-F	TTF1	c.836delA	p.K279Rfs*144	chr9:135277373	chr9:135277373	frameshift	9.09
P17-F	BAX	c.121delG	p.E41Rfs*19	chr19:49458971	chr19:49458971	frameshift	14.62
P17-F	MLH1	c.1348delG	p.D450Ifs*41	chr3:37067433	chr3:37067433	frameshift	11.63
P17-F	RUNX1T1	c.236delC	p.P79Qfs*11	chr8:93026928	chr8:93026928	frameshift	19.67
P17-F	CHD8	c.5607delC	p.D1870Tfs*21	chr14:21862347	chr14:21862347	frameshift	14.42
P17-F	MGMT	c.240delC	p.A81Lfs*10	chr10:131506176	chr10:131506176	frameshift	8.02
P17-F	RNF43	c.1976delG	p.G659Vfs*41	chr17:56435161	chr17:56435161	frameshift	17.50
P17-F	FGFR4	c.2298delC	p.S767Lfs*110	chr5:176524562	chr5:176524562	frameshift	17.48
P17-F	SLC3A2	c.899delA	p.K300Rfs*31	chr11:62649529	chr11:62649529	frameshift	16.67
P17-F	DICER1	c.4049delA	p.K1350Rfs*29	chr14:95569684	chr14:95569684	frameshift	15.91
P17-F	ARID1A	c.3975_3977delCCC	p.P1326del	chr1:27100176	chr1:27100178	inframe deletion	15.86
P17-F	STAT3	c.1847_1849delAAG	p.E616del	chr17:40475061	chr17:40475063	inframe deletion	9.41
P17-F	CHD4	c.458A>G	p.Q153R	chr12:6710913	chr12:6710913	missense	17.65

P17-F	APC	c.8140C>T	p.R2714C	chr5:112179431	chr5:112179431	missense	16.42
P17-F	SMARCA4	c.1765G>A	p.A589T	chr19:11107173	chr19:11107173	missense	8.00
P17-F	FAT1	c.7366G>A	p.A2456T	chr4:187540374	chr4:187540374	missense	16.07
P17-F	PALB2	c.3319C>A	p.L1107M	chr16:23619216	chr16:23619216	missense	13.24
P17-F	POLD1	c.607G>C	p.G203R	chr19:50905479	chr19:50905479	missense	16.67
P17-F	EXT1	c.122G>A	p.S41N	chr8:119123164	chr8:119123164	missense	3.76
P17-F	RB1	c.1849G>T	p.G617C	chr13:49030374	chr13:49030374	missense	8.93
P17-F	NOTCH1	c.6584G>A	p.G2195D	chr9:139391607	chr9:139391607	missense	16.49
P17-F	ERCC5	c.127C>T	p.R43W	chr13:103504506	chr13:103504506	missense	17.65
P17-F	LRP1B	c.2141C>T	p.A714V	chr2:141773314	chr2:141773314	missense	11.76
P17-F	SMARCA4	c.2561A>G	p.N854S	chr19:11130322	chr19:11130322	missense	10.26
P17-F	POLE	c.527A>G	p.K176R	chr12:133256134	chr12:133256134	missense	12.10
P17-F	MLH3	c.619G>A	p.V207I	chr14:75515740	chr14:75515740	missense	18.18
P17-F	NKX2-1	c.478C>T	p.P160S	chr14:36987121	chr14:36987121	missense	8.13
P17-F	LHCGR	c.50T>A	p.L17Q	chr2:48982761	chr2:48982761	missense	11.63
P17-F	SMAD4	c.1592G>A	p.R531Q	chr18:48604770	chr18:48604770	missense	8.77
P17-F	IRF2	c.146A>G	p.E49G	chr4:185340664	chr4:185340664	missense	15.07
P17-F	ASXL1	c.3090C>G	p.D1030E	chr20:31023605	chr20:31023605	missense	5.93
P17-F	SMARCB1	c.119G>A	p.R40Q	chr22:24133968	chr22:24133968	missense	14.75
P17-F	WRN	c.3974T>C	p.V1325A	chr8:31015038	chr8:31015038	missense	13.73
P17-F	EPAS1	c.541C>T	p.R181C	chr2:46587863	chr2:46587863	missense	12.20
P17-F	WAS	c.1097G>A	p.G366D	chrX:48547214	chrX:48547214	missense	10.16
P17-F	CTCF	c.1886C>T	p.A629V	chr16:67670641	chr16:67670641	missense	18.13
P17-F	PALB2	c.1808T>C	p.L603P	chr16:23641667	chr16:23641667	missense	15.79
P17-F	PARP1	c.2315A>G	p.E772G	chr1:226555272	chr1:226555272	missense	35.59
P17-F	BRCA2	c.2959A>G	p.N987D	chr13:32911451	chr13:32911451	missense	25.71
P17-F	RNF43	c.47T>C	p.L16P	chr17:56492892	chr17:56492892	missense	10.19
P17-F	THADA	c.2645C>T	p.A882V	chr2:43783513	chr2:43783513	missense	27.66
P17-F	ASXL1	c.2775G>T	p.Q925H	chr20:31023290	chr20:31023290	missense	20.22
P17-F	GSTP1	c.583G>A	p.A195T	chr11:67353998	chr11:67353998	missense	9.02
P17-F	TAP2	c.880C>A	p.L294I	chr6:32802996	chr6:32802996	missense	6.72
P17-F	NTRK3	c.1466C>T	p.T489I	chr15:88576207	chr15:88576207	missense	4.26
P17-F	BARD1	c.568G>A	p.D190N	chr2:215646030	chr2:215646030	missense	20.34
P17-F	DLL3	c.1606T>C	p.S536P	chr19:39998191	chr19:39998191	missense	8.08
P17-F	ATR	c.386C>T	p.S129L	chr3:142281858	chr3:142281858	missense	22.58
P17-F	RUNX1T1	c.1528C>A	p.H510N	chr8:92972646	chr8:92972646	missense	8.28
P17-F	PRDM1	c.1426C>T	p.R476W	chr6:106553461	chr6:106553461	missense	17.68
P17-F	THADA	c.3620A>G	p.Q1207R	chr2:43732762	chr2:43732762	missense	11.76
P17-F	NTRK3	c.1583C>T	p.T528M	chr15:88576090	chr15:88576090	missense	8.63
P17-F	POLD1	c.2959delG	p.D987Tfs*58	chr19:50919866	chr19:50919866	splice acceptor	20.17
P17-F	TAP2	c.2T>A	p.M1?	chr6:32806009	chr6:32806009	start lost	16.67
P17-F	RNF43	c.394C>T	p.R132*	chr17:56440943	chr17:56440943	nonsense	10.17
P18-PLA	TP53	c.831T>A	p.C277*	chr17:7577107	chr17:7577107	nonsense	79.81
P18-PLA	APC	c.4312dupA	p.T1438Nfs*17	chr5:112175599	chr5:112175600	frameshift	77.76
P18-PLA	SF3B1	c.3800A>G	p.K1267R	chr2:198257142	chr2:198257142	missense	48.70
P18-PLA	IRF2	c.250G>A	p.A84T	chr4:185339800	chr4:185339800	missense	43.62
P18-PLA	GATA1	c.1103G>T	p.G368V	chrX:48652432	chrX:48652432	missense	44.21
P18-PLA	GRM8	c.1855C>T	p.R619C	chr7:126173581	chr7:126173581	missense	19.80
P18-PLA	NF1	c.8112A>T	p.Q2704H	chr17:29685639	chr17:29685639	missense	8.79
P18-PLA	ERBB4	c.743C>T	p.A248V	chr2:212587258	chr2:212587258	missense	1.39

P18-PLA	AXIN2	c.1972A>T	p.S658C	chr17:63532607	chr17:63532607	missense	0.99
P18-PLA	IGF1R	c.335T>A	p.F112Y	chr15:99251031	chr15:99251031	missense	0.62
P2-F	TGFBR2	c.382_383delAA	p.K128Afs*3	chr3:30691872	chr3:30691873	frameshift	12.80
P2-F	KDM5A	c.3597delA	p.G1200Dfs*9	chr12:416953	chr12:416953	frameshift	6.41
P2-F	GATA3	c.708delC	p.S237Afs*29	chr10:8100728	chr10:8100728	frameshift	7.33
P2-F	SMARCA4	c.326delC	p.P109Rfs*194	chr19:11096048	chr19:11096048	frameshift	8.62
P2-F	MAP3K4	c.531delA	p.K177Nfs*30	chr6:161469829	chr6:161469829	frameshift	5.15
P2-F	SMARCA4	c.1079delG	p.G360Afs*51	chr19:11098558	chr19:11098558	frameshift	8.07
P2-F	JARID2	c.2028delA	p.K676Nfs*5	chr6:15501215	chr6:15501215	frameshift	16.08
P2-F	FLT4	c.89dupC	p.T31Dfs*15	chr5:180058747	chr5:180058748	frameshift	8.52
P2-F	RNF43	c.1976delG	p.G659Vfs*41	chr17:56435161	chr17:56435161	frameshift	15.04
P2-F	BRCA2	c.1813delA	p.I605Yfs*9	chr13:32907421	chr13:32907421	frameshift	5.35
P2-F	QKI	c.401delA	p.K134Rfs*14	chr6:163899920	chr6:163899920	frameshift	6.44
P2-F	ATR	c.2320dupA	p.I774Nfs*3	chr3:142274739	chr3:142274740	frameshift	7.92
P2-F	ETV4	c.399dupC	p.R134Qfs*49	chr17:41610700	chr17:41610701	frameshift	6.30
P2-F	NBN	c.1396delA	p.R466Gfs*18	chr8:90967512	chr8:90967512	frameshift	7.58
P2-F	PRDM1	c.1136C>T	p.A379V	chr6:106553171	chr6:106553171	missense	8.12
P2-F	STAT3	c.2186G>A	p.R729H	chr17:40468878	chr17:40468878	missense	6.14
P2-F	MAP2K2	c.793T>C	p.Y265H	chr19:4099325	chr19:4099325	missense	7.36
P2-F	CEBPA	c.125C>A	p.P42H	chr19:33793196	chr19:33793196	missense	9.60
P2-F	KDM5A	c.3179C>T	p.T1060M	chr12:420088	chr12:420088	missense	7.74
P2-F	RECQL4	c.2063T>C	p.L688P	chr8:145739092	chr8:145739092	missense	7.47
P2-F	PGR	c.1283G>A	p.R428Q	chr11:100998519	chr11:100998519	missense	7.74
P2-F	PIK3R2	c.2039C>T	p.A680V	chr19:18279956	chr19:18279956	missense	9.35
P2-F	PAK3	c.50G>T	p.R17M	chrX:110366381	chrX:110366381	missense	6.65
P2-F	MED12	c.5711C>T	p.A1904V	chrX:70357196	chrX:70357196	missense	7.41
P2-F	NSD1	c.2140G>T	p.G714C	chr5:176637540	chr5:176637540	missense	8.30
P2-F	SMARCA4	c.3032T>C	p.M1011T	chr19:11135065	chr19:11135065	missense	2.41
P2-F	MED12	c.2303G>A	p.R768H	chrX:70345277	chrX:70345277	missense	7.37
P2-F	EPHA5	c.701C>A	p.P234H	chr4:66467568	chr4:66467568	missense	7.54
P2-F	GNAS	c.601C>T	p.R201C	chr20:57484420	chr20:57484420	missense	7.17
P2-F	KMT2B	c.3292G>A	p.G1098R	chr19:36214866	chr19:36214866	missense	8.05
P2-F	MET	c.3166A>C	p.S1056R	chr7:116415072	chr7:116415072	missense	6.10
P2-F	MAP2K2	c.660_661delCGinsAT	p.D221Y	chr19:4101061	chr19:4101062	missense	8.92
P2-F	CYP2D6	c.701A>G	p.N234S	chr22:42523975	chr22:42523975	missense	5.72
P2-F	NF1	c.6806G>A	p.R2269H	chr17:29665144	chr17:29665144	missense	5.57
P2-F	AXL	c.2276T>C	p.L759P	chr19:41763504	chr19:41763504	missense	8.98
P2-F	SETBP1	c.2210C>T	p.P737L	chr18:42531515	chr18:42531515	missense	6.98
P2-F	KMT2A	c.9212T>C	p.L3071P	chr11:118375828	chr11:118375828	missense	7.03
P2-F	NOTCH1	c.448G>A	p.A150T	chr9:139417596	chr9:139417596	missense	2.72
P2-F	POLE	c.5035C>T	p.R1679C	chr12:133218901	chr12:133218901	missense	7.17
P2-F	FGFR4	c.892G>A	p.G298S	chr5:176519486	chr5:176519486	missense	8.08
P2-F	TSC2	c.3815-1G>A	-	chr16:2132436	chr16:2132436	splice acceptor	45.92
P2-F	LZTR1	c.994-2A>G	-	chr22:21346501	chr22:21346501	splice acceptor	7.68
P2-F	MGMT	c.218+2T>C	-	chr10:131334643	chr10:131334643	splice donor	9.28
P2-F	APC	c.3880C>T	p.Q1294*	chr5:112175171	chr5:112175171	nonsense	6.13
P3-T	MSH6	c.3261dupC	p.F1088Lfs*5	chr2:48030639	chr2:48030640	frameshift	15.44
P3-T	DENND1A	c.2456delC	p.P819Lfs*124	chr9:126144285	chr9:126144285	frameshift	7.54
P3-T	MLLT3	c.892delA	p.R298Gfs*19	chr9:20413952	chr9:20413952	frameshift	5.77
P3-T	KDM5A	c.3597delA	p.G1200Dfs*9	chr12:416953	chr12:416953	frameshift	7.25

P3-T	AXL	c.874dupC	p.H292Pfs*47	chr19:41743932	chr19:41743933	frameshift	16.87
P3-T	WRN	c.15delA	p.K5Nfs*15	chr8:30915971	chr8:30915971	frameshift	16.22
P3-T	CDH1	c.1779delC	p.I594Yfs*19	chr16:68855967	chr16:68855967	frameshift	36.93
P3-T	CHEK2	c.64dupC	p.H22Pfs*55	chr22:29130645	chr22:29130646	frameshift	18.49
P3-T	MAP3K4	c.479delA	p.N160Mfs*8	chr6:161469776	chr6:161469776	frameshift	7.60
P3-T	SETBP1	c.640delA	p.S214Afs*129	chr18:42529940	chr18:42529940	frameshift	6.55
P3-T	PBRM1	c.835dupA	p.I279Nfs*8	chr3:52678783	chr3:52678784	frameshift	12.35
P3-T	EPAS1	c.475delA	p.S159Afs*12	chr2:46587791	chr2:46587791	frameshift	13.45
P3-T	RNF43	c.1976delG	p.G659Vfs*41	chr17:56435161	chr17:56435161	frameshift	18.55
P3-T	NBN	c.1651delA	p.R551Gfs*8	chr8:90965666	chr8:90965666	frameshift	8.33
P3-T	APC	c.5612_5614dupATG	p.D1871dup	chr5:112176891	chr5:112176892	inframe insertion	12.68
P3-T	ROS1	c.260C>T	p.A87V	chr6:117730774	chr6:117730774	missense	6.62
P3-T	ARAF	c.988C>T	p.H330Y	chrX:47426743	chrX:47426743	missense	22.13
P3-T	RUNX1	c.1424C>T	p.A475V	chr21:36164451	chr21:36164451	missense	14.80
P3-T	ERBB3	c.536A>G	p.N179S	chr12:56480429	chr12:56480429	missense	21.10
P3-T	CTNNB1	c.1355T>C	p.L452P	chr3:41275189	chr3:41275189	missense	17.72
P3-T	YAP1	c.589A>C	p.T197P	chr11:102033203	chr11:102033203	missense	9.41
P3-T	FGFR2	c.16C>T	p.R6C	chr10:123353316	chr10:123353316	missense	16.46
P3-T	ERBB4	c.2828C>T	p.P943L	chr2:212288918	chr2:212288918	missense	14.59
P3-T	SOX2	c.899G>A	p.S300N	chr3:181431047	chr3:181431047	missense	29.39
P3-T	TUBB3	c.982G>A	p.E328K	chr16:90001841	chr16:90001841	missense	2.39
P3-T	ARID2	c.3544T>C	p.F1182L	chr12:46245450	chr12:46245450	missense	8.58
P3-T	AR	c.758C>T	p.A253V	chrX:66765746	chrX:66765746	missense	17.36
P3-T	VEGFA	c.1227G>T	p.K409N	chr6:43752287	chr6:43752287	missense	8.24
P3-T	PRF1	c.199C>A	p.L67M	chr10:72360460	chr10:72360460	missense	5.89
P3-T	EPHA2	c.508G>A	p.V170M	chr1:16475188	chr1:16475188	missense	2.56
P3-T	AXIN2	c.1795G>C	p.A599P	chr17:63533099	chr17:63533099	missense	18.47
P3-T	PDE11A	c.1486C>T	p.R496C	chr2:178704992	chr2:178704992	missense	8.46
P3-T	CSF1R	c.2806G>T	p.G936C	chr5:149433745	chr5:149433745	missense	17.69
P3-T	PTCH1	c.2126G>T	p.R709M	chr9:98231157	chr9:98231157	missense	17.54
P3-T	ATRX	c.1492A>G	p.R498G	chrX:76939256	chrX:76939256	missense	2.46
P3-T	KMT2A	c.6223C>T	p.R2075C	chr11:118371775	chr11:118371775	missense	19.08
P3-T	MUTYH	c.229T>G	p.S77A	chr1:45799195	chr1:45799195	missense	19.27
P3-T	AMER1	c.1249C>T	p.P417S	chrX:63411918	chrX:63411918	missense	20.57
P3-T	TGFBR2	c.1150A>G	p.N384D	chr3:30713825	chr3:30713825	missense	9.14
P3-T	NSD1	c.3518G>A	p.R1173H	chr5:176638918	chr5:176638918	missense	21.74
P3-T	CBLB	c.875T>C	p.L292S	chr3:105459446	chr3:105459446	missense	14.68
P3-T	POLE	c.4046C>T	p.A1349V	chr12:133225618	chr12:133225618	missense	2.01
P3-T	FOXA1	c.326G>T	p.S109I	chr14:38061663	chr14:38061663	missense	20.99
P3-T	ATM	c.4A>G	p.S2G	chr11:108098355	chr11:108098355	missense	19.75
P3-T	PBRM1	c.4046A>G	p.H1349R	chr3:52595869	chr3:52595869	missense	6.02
P3-T	TGFBR2	c.689C>T	p.T230M	chr3:30713364	chr3:30713364	missense	17.25
P3-T	TYMS	c.5C>A	p.P2H	chr18:657747	chr18:657747	missense	24.02
P3-T	BTG2	c.440G>A	p.S147N	chr1:203276529	chr1:203276529	missense	17.38
P3-T	MTOR	c.4135G>T	p.G1379C	chr1:11259433	chr1:11259433	missense	18.72
P3-T	PRKCI	c.773G>T	p.R258L	chr3:169998082	chr3:169998082	missense	6.65
P3-T	KDM5A	c.3397C>T	p.R1133W	chr12:417153	chr12:417153	missense	8.57
P3-T	TP53	c.818G>A	p.R273H	chr17:7577120	chr17:7577120	missense	20.25
P3-T	PGR	c.1205G>A	p.R402H	chr11:100998597	chr11:100998597	missense	17.26
P3-T	C11orf30	c.3628T>C	p.S1210P	chr11:76257195	chr11:76257195	missense	18.30

P3-T	POLE	c.6049C>T	p.R2017C	chr12:133209337	chr12:133209337	missense	18.47
P3-T	DDR2	c.92G>A	p.R31H	chr1:162722894	chr1:162722894	missense	11.44
P3-T	FANCE	c.518T>C	p.L173P	chr6:35423793	chr6:35423793	missense	2.58
P3-T	RRM1	c.1496G>A	p.R499H	chr11:4148290	chr11:4148290	missense	9.17
P3-T	ALK	c.2485A>G	p.K829E	chr2:29456433	chr2:29456433	missense	6.82
P3-T	CYP2A7	c.971A>T	p.E324V	chr19:41383759	chr19:41383759	missense	2.01
P3-T	FLT3	c.2858C>T	p.A953V	chr13:28588590	chr13:28588590	missense	18.73
P3-T	POLD1	c.2959delG	p.D987Tfs*58	chr19:50919866	chr19:50919866	splice acceptor	9.50
P3-T	CD274	c.-14-2A>G	-	chr9:5456098	chr9:5456098	splice acceptor	6.36
P3-T	POLE	c.2554C>T	p.Q852*	chr12:133240963	chr12:133240963	nonsense	18.84
P3-T	CXCR4	c.755G>A	p.W252*	chr2:136872743	chr2:136872743	nonsense	15.70
P4-F	NF1	c.7318G>T	p.A2440S	chr17:29676266	chr17:29676266	missense	3.33
P4-F	ROS1	c.6805C>T	p.R2269*	chr6:117609894	chr6:117609894	nonsense	23.25
P5-F	PDE11A	c.498delC	p.T167Pfs*14	chr2:178936667	chr2:178936667	frameshift	22.08
P5-F	FLCN	c.1285delC	p.H429Tfs*39	chr17:17119709	chr17:17119709	frameshift	9.21
P5-F	RNF43	c.1976delG	p.G659Vfs*41	chr17:56435161	chr17:56435161	frameshift	38.23
P5-F	MLH3	c.1755delA	p.E586Nfs*24	chr14:75514604	chr14:75514604	frameshift	21.34
P5-F	ARID1A	c.3344delC	p.P1115Qfs*46	chr1:27097751	chr1:27097751	frameshift	19.37
P5-F	PDCD1	c.105delC	p.T36Pfs*9	chr2:242795104	chr2:242795104	frameshift	7.02
P5-F	FANCA	c.1615delG	p.D539Tfs*66	chr16:89849278	chr16:89849278	frameshift	2.70
P5-F	RNF43	c.349dupC	p.R117Pfs*8	chr17:56448297	chr17:56448298	frameshift	18.67
P5-F	PRKCI	c.826dupA	p.T276Nfs*16	chr3:169998127	chr3:169998128	frameshift	16.63
P5-F	SRC	c.1066delG	p.E356Rfs*28	chr20:36030027	chr20:36030027	frameshift	18.90
P5-F	JARID2	c.1272dupG	p.R425Afs*99	chr6:15496721	chr6:15496722	frameshift	14.89
P5-F	ATM	c.640delT	p.S214Pfs*16	chr11:108114817	chr11:108114817	frameshift	17.01
P5-F	DNMT3A	c.2727delT	p.F909Lfs*13	chr2:25457160	chr2:25457160	frameshift	2.63
P5-F	TEK	c.255delA	p.V86Lfs*31	chr9:27158026	chr9:27158026	frameshift	18.64
P5-F	BAX	c.121dupG	p.E41Gfs*33	chr19:49458970	chr19:49458971	frameshift	14.74
P5-F	KMT2B	c.1656delC	p.K553Nfs*52	chr19:36211899	chr19:36211899	frameshift	17.61
P5-F	RAD50	c.2165dupA	p.E723Gfs*5	chr5:131931451	chr5:131931452	frameshift	10.55
P5-F	WRN	c.3382delA	p.S1128Vfs*34	chr8:31001132	chr8:31001132	frameshift	16.39
P5-F	AR	c.1359delT	p.G455Vfs*24	chrX:66766347	chrX:66766347	frameshift	3.03
P5-F	SLC3A2	c.899delA	p.K300Rfs*31	chr11:62649529	chr11:62649529	frameshift	20.06
P5-F	CREBBP	c.3250delA	p.I1084Sfs*15	chr16:3817721	chr16:3817721	frameshift	17.98
P5-F	CCND1	c.822_824dupGGA	p.E280dup	chr11:69465975	chr11:69465976	inframe insertion	14.21
P5-F	PLK1	c.512G>A	p.R171Q	chr16:23691508	chr16:23691508	missense	19.45
P5-F	SOX2	c.479A>G	p.Y160C	chr3:181430627	chr3:181430627	missense	2.21
P5-F	MTOR	c.1405G>A	p.A469T	chr1:11303178	chr1:11303178	missense	14.95
P5-F	KDM5A	c.4162A>G	p.T1388A	chr12:406279	chr12:406279	missense	20.18
P5-F	FBXW7	c.1393C>T	p.R465C	chr4:153249385	chr4:153249385	missense	19.07
P5-F	NBN	c.546A>C	p.K182N	chr8:90990486	chr8:90990486	missense	18.42
P5-F	PIK3CA	c.2008C>T	p.H670Y	chr3:178937833	chr3:178937833	missense	3.03
P5-F	FLT1	c.2839C>T	p.P947S	chr13:28897041	chr13:28897041	missense	16.56
P5-F	EPHA3	c.2525G>A	p.R842Q	chr3:89499355	chr3:89499355	missense	19.92
P5-F	NOTCH2	c.1619C>T	p.P540L	chr1:120508138	chr1:120508138	missense	18.36
P5-F	FANCC	c.1486C>T	p.L496F	chr9:97869395	chr9:97869395	missense	3.28
P5-F	C11orf30	c.2653G>A	p.V885M	chr11:76253355	chr11:76253355	missense	19.36
P5-F	CBLB	c.908A>G	p.D303G	chr3:105459413	chr3:105459413	missense	21.61
P5-F	TTF1	c.676T>A	p.S226T	chr9:135277533	chr9:135277533	missense	2.21
P5-F	BRCA2	c.8971C>T	p.R2991C	chr13:32953904	chr13:32953904	missense	17.37

P5-F	KMT2B	c.6581T>C	p.V2194A	chr19:36224031	chr19:36224031	missense	2.00
P5-F	MAP3K1	c.2419A>G	p.N807D	chr5:56177446	chr5:56177446	missense	3.26
P5-F	JAK2	c.2446T>G	p.L816V	chr9:5081736	chr9:5081736	missense	23.73
P5-F	MAP3K4	c.319C>T	p.P107S	chr6:161455457	chr6:161455457	missense	2.70
P5-F	THADA	c.251G>A	p.G84D	chr2:43818014	chr2:43818014	missense	2.17
P5-F	NOTCH1	c.4070G>A	p.C1357Y	chr9:139400278	chr9:139400278	missense	19.01
P5-F	PKHD1	c.8380G>A	p.D2794N	chr6:51656094	chr6:51656094	missense	3.08
P5-F	FGFR4	c.608G>A	p.R203H	chr5:176518690	chr5:176518690	missense	22.20
P5-F	SLC34A2	c.271A>T	p.I91F	chr4:25665844	chr4:25665844	missense	2.13
P5-F	NKX2-1	c.87G>A	p.M29I	chr14:36988476	chr14:36988476	missense	17.77
P5-F	KMT2B	c.3596G>C	p.G1199A	chr19:36216188	chr19:36216188	missense	22.24
P5-F	MSH2	c.2662C>A	p.L888M	chr2:47709945	chr2:47709945	missense	2.35
P5-F	STMN1	c.160T>A	p.L54I	chr1:26230158	chr1:26230158	missense	2.48
P5-F	CREBBP	c.4990C>T	p.R1664C	chr16:3781375	chr16:3781375	missense	18.14
P5-F	AXIN2	c.855A>G	p.I285M	chr17:63545739	chr17:63545739	missense	3.18
P5-F	NKX2-1	c.697A>G	p.S233G	chr14:36986902	chr14:36986902	missense	13.72
P5-F	MAP3K1	c.2518A>G	p.M840V	chr5:56177545	chr5:56177545	missense	2.07
P5-F	VHL	c.103G>A	p.A35T	chr3:10183634	chr3:10183634	missense	17.04
P5-F	KMT2A	c.5255G>A	p.S1752N	chr11:118365088	chr11:118365088	missense	4.19
P5-F	ERCC5	c.1537G>A	p.A513T	chr13:103515036	chr13:103515036	missense	17.19
P5-F	CHEK1	c.898T>G	p.F300V	chr11:125513770	chr11:125513770	missense	2.98
P5-F	EPHA3	c.2283G>T	p.K761N	chr3:89480446	chr3:89480446	missense	18.15
P5-F	DDR2	c.470C>T	p.P157L	chr1:162724998	chr1:162724998	missense	21.39
P5-F	PIK3C3	c.1747A>T	p.M583L	chr18:39613829	chr18:39613829	missense	2.29
P5-F	RAF1	c.1841C>A	p.P614Q	chr3:12626119	chr3:12626119	missense	3.70
P5-F	CDH1	c.1307T>C	p.L436S	chr16:68847385	chr16:68847385	missense	2.41
P5-F	ARID1A	c.5045C>T	p.A1682V	chr1:27102119	chr1:27102119	missense	3.38
P5-F	TP63	c.415G>A	p.A139T	chr3:189526151	chr3:189526151	missense	17.36
P5-F	MED12	c.593T>C	p.L198S	chrX:70340860	chrX:70340860	missense	5.28
P5-F	SMO	c.1136C>T	p.A379V	chr7:128846206	chr7:128846206	missense	17.73
P5-F	NKX2-1	c.836C>T	p.A279V	chr14:36986763	chr14:36986763	missense	9.36
P5-F	KMT2B	c.2117G>A	p.R706Q	chr19:36212366	chr19:36212366	missense	21.60
P5-F	PDGFRA	c.701C>T	p.T234I	chr4:55131158	chr4:55131158	missense	2.41
P5-F	FAT1	c.13040C>A	p.P4347H	chr4:187516941	chr4:187516941	missense	2.87
P5-F	PALB2	c.101G>A	p.R34H	chr16:23649398	chr16:23649398	missense	2.60
P5-F	PIK3R2	c.1954C>T	p.R652W	chr19:18279681	chr19:18279681	missense	20.03
P5-F	RET	c.206G>A	p.G69D	chr10:43596039	chr10:43596039	missense	20.34
P5-F	CREBBP	c.1868A>C	p.D623A	chr16:3828774	chr16:3828774	missense	2.72
P5-F	IRF2	c.236C>T	p.A79V	chr4:185339814	chr4:185339814	missense	15.71
P5-F	CBL	c.1296T>A	p.D432E	chr11:119149288	chr11:119149288	missense	23.06
P5-F	STAT3	c.2186G>A	p.R729H	chr17:40468878	chr17:40468878	missense	16.91
P5-F	RET	c.682G>A	p.A228T	chr10:43600456	chr10:43600456	missense	8.92
P5-F	CHD4	c.4309G>T	p.D1437Y	chr12:6691842	chr12:6691842	missense	2.58
P5-F	CDKN2A	c.286G>A	p.V96M	chr9:21971072	chr9:21971072	missense	19.96
P5-F	THADA	c.2579C>A	p.A860D	chr2:43783579	chr2:43783579	missense	2.38
P5-F	JARID2	c.1025T>C	p.V342A	chr6:15496481	chr6:15496481	missense	7.19
P5-F	TNFAIP3	c.1340C>T	p.A447V	chr6:138199922	chr6:138199922	missense	17.31
P5-F	CRKL	c.853C>A	p.L285I	chr22:21304074	chr22:21304074	missense	3.61
P5-F	RRM1	c.434A>G	p.Y145C	chr11:4130920	chr11:4130920	missense	9.04
P5-F	BAI3	c.3641G>A	p.G1214E	chr6:70070806	chr6:70070806	missense	17.55

P5-F	GNAS	c.992C>T	p.A331V	chr20:57429312	chr20:57429312	missense	21.32
P5-F	KDR	c.3990A>G	p.I1330M	chr4:55946189	chr4:55946189	missense	4.73
P5-F	NRG1	c.279-1G>A	-	chr8:32463079	chr8:32463079	splice acceptor	24.13
P5-F	NFKBIA	c.227+1G>A	-	chr14:35873623	chr14:35873623	splice donor	2.94
P5-F	DUSP2	c.385C>T	p.R129*	chr2:96810709	chr2:96810709	nonsense	2.30
P5-F	PLK1	c.511C>T	p.R171*	chr16:23691507	chr16:23691507	nonsense	18.46
P5-F	APC	c.3139G>T	p.E1047*	chr5:112174430	chr5:112174430	nonsense	18.23
P6-F	WISP3	c.678delA	p.K226Nfs*24	chr6:112389434	chr6:112389434	frameshift	24.68
P6-F	SETD2	c.843delA	p.E282Kfs*19	chr3:47165283	chr3:47165283	frameshift	20.68
P6-F	AXL	c.1010delC	p.P337Lfs*29	chr19:41744385	chr19:41744385	frameshift	22.02
P6-F	ALK	c.4796delC	p.P1599Lfs*11	chr2:29416157	chr2:29416157	frameshift	20.16
P6-F	CHD4	c.218dupA	p.E74Gfs*33	chr12:6711545	chr12:6711546	frameshift	17.84
P6-F	KMT2B	c.4235delG	p.G1412Afs*10	chr19:36218451	chr19:36218451	frameshift	26.45
P6-F	RNF43	c.349dupC	p.R117Pfs*8	chr17:56448297	chr17:56448298	frameshift	45.69
P6-F	KMT2B	c.6678delC	p.T2227Rfs*34	chr19:36224124	chr19:36224124	frameshift	25.48
P6-F	PALB2	c.886delA	p.M296*	chr16:23646981	chr16:23646981	frameshift	18.74
P6-F	RUNX1T1	c.236delC	p.P79Qfs*11	chr8:93026928	chr8:93026928	frameshift	15.14
P6-F	DLL3	c.1781delC	p.P594Rfs*24	chr19:39998572	chr19:39998572	frameshift	20.83
P6-F	SETD2	c.4219delA	p.R1407Gfs*5	chr3:47161907	chr3:47161907	frameshift	20.83
P6-F	SLC3A2	c.899delA	p.K300Rfs*31	chr11:62649529	chr11:62649529	frameshift	24.17
P6-F	ARID1A	c.5548delG	p.D1850Tfs*33	chr1:27105931	chr1:27105931	frameshift	17.56
P6-F	EP300	c.4408delA	p.M1470Cfs*26	chr22:41566525	chr22:41566525	frameshift	21.32
P6-F	PKHD1	c.2119_2121delATT	p.I707del	chr6:51917893	chr6:51917895	inframe deletion	18.37
P6-F	CSF1R	c.467_469delTCT	p.F156del	chr5:149459738	chr5:149459740	inframe deletion	19.11
P6-F	SDHA	c.991G>A	p.A331T	chr5:233687	chr5:233687	missense	18.96
P6-F	ESR1	c.109C>T	p.R37W	chr6:152129156	chr6:152129156	missense	3.13
P6-F	ARID1B	c.6355A>G	p.M2119V	chr6:157528669	chr6:157528669	missense	2.95
P6-F	TGFBR2	c.1583G>A	p.R528H	chr3:30732970	chr3:30732970	missense	23.38
P6-F	STAT3	c.2186G>A	p.R729H	chr17:40468878	chr17:40468878	missense	25.90
P6-F	TSC2	c.4145T>G	p.L1382R	chr16:2134368	chr16:2134368	missense	25.00
P6-F	CYP2A6	c.376C>G	p.Q126E	chr19:41354636	chr19:41354636	missense	17.59
P6-F	KMT2A	c.5411A>G	p.Q1804R	chr11:118366471	chr11:118366471	missense	19.79
P6-F	ERCC3	c.995G>A	p.R332H	chr2:128046268	chr2:128046268	missense	16.90
P6-F	EWSR1	c.692G>A	p.S231N	chr22:29683022	chr22:29683022	missense	21.58
P6-F	IKBKE	c.1327C>A	p.L443M	chr1:206653443	chr1:206653443	missense	19.81
P6-F	PIK3CA	c.1162A>G	p.N388D	chr3:178927399	chr3:178927399	missense	18.86
P6-F	INPP4B	c.2711A>G	p.Q904R	chr4:142949999	chr4:142949999	missense	18.98
P6-F	DAXX	c.1849G>A	p.V617I	chr6:33287248	chr6:33287248	missense	19.66
P6-F	CYP2A6	c.443G>A	p.R148H	chr19:41354569	chr19:41354569	missense	21.97
P6-F	TSC2	c.2584G>A	p.A862T	chr16:2125838	chr16:2125838	missense	21.33
P6-F	ERCC3	c.1358G>T	p.R453M	chr2:128038192	chr2:128038192	missense	2.00
P6-F	AKT3	c.70C>T	p.P24S	chr1:243858995	chr1:243858995	missense	2.22
P6-F	PDGFRA	c.2134C>A	p.P712T	chr4:55144660	chr4:55144660	missense	21.47
P6-F	GRM8	c.1697G>A	p.R566H	chr7:126173739	chr7:126173739	missense	22.84
P6-F	ATR	c.1963G>C	p.V655L	chr3:142275340	chr3:142275340	missense	21.55
P6-F	WRN	c.1679T>C	p.V560A	chr8:30948007	chr8:30948007	missense	13.84
P6-F	RAF1	c.1771A>G	p.K591E	chr3:12626378	chr3:12626378	missense	23.95
P6-F	SMARCA4	c.4532A>C	p.K1511T	chr19:11169038	chr19:11169038	missense	21.82
P6-F	NOTCH2	c.2219+2T>C	-	chr1:120497661	chr1:120497661	splice donor	17.74
P6-F	POLE	c.4210G>T	p.E1404*	chr12:133220503	chr12:133220503	nonsense	19.01

P7-F	MSH6	c.3261dupC	p.F1088Lfs*5	chr2:48030639	chr2:48030640	frameshift	12.02
P7-F	RNF43	c.1468delG	p.V490Sfs*12	chr17:56435669	chr17:56435669	frameshift	12.27
P7-F	RECQL4	c.2637delC	p.S880Qfs*?	chr8:145738349	chr8:145738349	frameshift	8.97
P7-F	SUFU	c.71delC	p.P24Rfs*72	chr10:104263974	chr10:104263974	frameshift	17.94
P7-F	EPHA2	c.987delC	p.S330Pfs*63	chr1:16464673	chr1:16464673	frameshift	11.12
P7-F	KDM5A	c.3597delA	p.G1200Dfs*9	chr12:416953	chr12:416953	frameshift	13.15
P7-F	NF1	c.5878delT	p.C1960Afs*19	chr17:29661917	chr17:29661917	frameshift	8.91
P7-F	POLE	c.6084_6085insA	p.G2029Rfs*30	chr12:133209301	chr12:133209302	frameshift	11.37
P7-F	MEF2B	c.819delC	p.T274Pfs*130	chr19:19257144	chr19:19257144	frameshift	14.33
P7-F	ARID2	c.5165dupG	p.T1723Hfs*6	chr12:46287215	chr12:46287216	frameshift	13.86
P7-F	TGFBR2	c.382_383delAA	p.K128Afs*3	chr3:30691872	chr3:30691873	frameshift	16.32
P7-F	BAX	c.121delG	p.E41Rfs*19	chr19:49458971	chr19:49458971	frameshift	11.86
P7-F	FANCM	c.4005delA	p.V1336Lfs*2	chr14:45645955	chr14:45645955	frameshift	13.21
P7-F	KDM5A	c.3597dupA	p.G1200Rfs*7	chr12:416952	chr12:416953	frameshift	7.06
P7-F	FGFR3	c.1206dupC	p.K403Qfs*93	chr4:1806180	chr4:1806181	frameshift	13.93
P7-F	RAD50	c.2165dupA	p.E723Gfs*5	chr5:131931451	chr5:131931452	frameshift	8.39
P7-F	CHEK1	c.676dupA	p.T226Nfs*19	chr11:125505377	chr11:125505378	frameshift	9.69
P7-F	RNF43	c.1976delG	p.G659Vfs*41	chr17:56435161	chr17:56435161	frameshift	25.46
P7-F	KMT2B	c.3596delG	p.G1199Afs*156	chr19:36216184	chr19:36216184	frameshift	14.14
P7-F	BLM	c.1544delA	p.N515Mfs*16	chr15:91304139	chr15:91304139	frameshift	12.12
P7-F	DUSP2	c.757delG	p.V253Cfs*130	chr2:96809750	chr2:96809750	frameshift	15.03
P7-F	SLC3A2	c.899delA	p.K300Rfs*31	chr11:62649529	chr11:62649529	frameshift	15.72
P7-F	CREBBP	c.3250delA	p.I1084Sfs*15	chr16:3817721	chr16:3817721	frameshift	13.57
P7-F	ARID1A	c.3999_4001delGCA	p.Q1334del	chr1:27100182	chr1:27100184	inframe deletion	13.65
P7-F	TOP2A	c.2907_2909delTGT	p.V970del	chr17:38556552	chr17:38556554	inframe deletion	16.40
P7-F	FBXW7	c.1393C>T	p.R465C	chr4:153249385	chr4:153249385	missense	10.94
P7-F	VHL	c.562C>A	p.L188M	chr3:10191569	chr3:10191569	missense	2.84
P7-F	KEAP1	c.1481G>T	p.R494M	chr19:10600374	chr19:10600374	missense	13.42
P7-F	PIK3R2	c.1435A>G	p.T479A	chr19:18276988	chr19:18276988	missense	13.21
P7-F	SETBP1	c.3407A>G	p.K1136R	chr18:42532712	chr18:42532712	missense	11.97
P7-F	FAT1	c.1654G>A	p.E552K	chr4:187629328	chr4:187629328	missense	12.14
P7-F	HNF1A	c.1450G>A	p.V484M	chr12:121435417	chr12:121435417	missense	11.89
P7-F	PTEN	c.140G>T	p.R47M	chr10:89653842	chr10:89653842	missense	13.08
P7-F	ERCC1	c.478C>A	p.L160M	chr19:45922403	chr19:45922403	missense	14.33
P7-F	NSD1	c.6851A>G	p.E2284G	chr5:176721220	chr5:176721220	missense	12.69
P7-F	GRIN2A	c.1569C>A	p.D523E	chr16:9934586	chr16:9934586	missense	12.11
P7-F	HNF1A	c.475C>T	p.R159W	chr12:121426784	chr12:121426784	missense	11.91
P7-F	KMT2B	c.7147C>T	p.H2383Y	chr19:36224761	chr19:36224761	missense	13.32
P7-F	KMT2B	c.8119G>A	p.A2707T	chr19:36229429	chr19:36229429	missense	11.25
P7-F	KDR	c.3125T>C	p.V1042A	chr4:55956190	chr4:55956190	missense	15.98
P7-F	CXCR4	c.89G>A	p.R30H	chr2:136873409	chr2:136873409	missense	14.79
P7-F	BRCA2	c.6100C>T	p.R2034C	chr13:32914592	chr13:32914592	missense	12.36
P7-F	TP53	c.524G>A	p.R175H	chr17:7578406	chr17:7578406	missense	14.74
P7-F	NOTCH2	c.4016G>T	p.G1339V	chr1:120468423	chr1:120468423	missense	13.61
P7-F	JAK3	c.181A>G	p.S61G	chr19:17955046	chr19:17955046	missense	14.95
P7-F	ZNF703	c.965C>T	p.A322V	chr8:37555384	chr8:37555384	missense	11.11
P7-F	MEF2B	c.122G>A	p.C41Y	chr19:19260171	chr19:19260171	missense	13.11
P7-F	KDR	c.2312C>T	p.T771M	chr4:55964925	chr4:55964925	missense	13.52
P7-F	C11orf30	c.2030C>T	p.A677V	chr11:76239346	chr11:76239346	missense	15.48
P7-F	FGFR3	c.335G>A	p.R112Q	chr4:1801206	chr4:1801206	missense	16.55

P7-F	CDKN2A	c.200G>A	p.G67D	chr9:21971158	chr9:21971158	missense	13.66
P7-F	SPRY4	c.458G>A	p.R153H	chr5:141694285	chr5:141694285	missense	11.99
P7-F	ERBB3	c.1208C>T	p.P403L	chr12:56486794	chr12:56486794	missense	14.67
P7-F	PGR	c.1403C>T	p.A468V	chr11:100998399	chr11:100998399	missense	9.94
P7-F	PPP2R1A	c.200T>C	p.L67P	chr19:52709246	chr19:52709246	missense	22.87
P7-F	TNFAIP3	c.1078A>G	p.S360G	chr6:138199660	chr6:138199660	missense	11.60
P7-F	CSF1R	c.2348A>G	p.N783S	chr5:149435876	chr5:149435876	missense	11.76
P7-F	ROS1	c.5999T>C	p.L2000P	chr6:117639357	chr6:117639357	missense	9.70
P7-F	IKBKE	c.1252G>A	p.V418M	chr1:206653368	chr1:206653368	missense	12.58
P7-F	BRCA2	c.472T>C	p.S158P	chr13:32900284	chr13:32900284	missense	9.52
P7-F	POLD1	c.932G>A	p.R311H	chr19:50905960	chr19:50905960	missense	12.97
P7-F	TSC2	c.3554C>T	p.A1185V	chr16:2130322	chr16:2130322	missense	12.13
P7-F	NTRK1	c.2242C>T	p.R748W	chr1:156851285	chr1:156851285	missense	10.87
P7-F	TP53	c.31G>A	p.E11K	chr17:7579882	chr17:7579882	missense	15.82
P7-F	PLK1	c.1194G>T	p.E398D	chr16:23699990	chr16:23699990	missense	13.11
P7-F	MAP2K4	c.219-1G>C	-	chr17:11984672	chr17:11984672	splice acceptor	13.10
P7-F	WAS	c.273+1G>A	-	chrX:48542813	chrX:48542813	splice donor	9.89
P7-F	MEN1	c.915C>T	-	chr11:64573838	chr11:64573838	splice region	11.91
P7-F	ARID1A	c.1345C>T	p.Q449*	chr1:27056349	chr1:27056349	nonsense	12.57
P7-F	EP300	c.1306G>T	p.G436*	chr22:41527415	chr22:41527415	nonsense	13.33
P8-F	SMARCA4	c.2858dupA	p.V954Gfs*5	chr19:11132638	chr19:11132639	frameshift	15.26
P8-F	CREBBP	c.6949delC	p.Q2317Sfs*79	chr16:3778099	chr16:3778099	frameshift	5.48
P8-F	MECOM	c.1839delA	p.G614Efs*30	chr3:168833257	chr3:168833257	frameshift	15.16
P8-F	QKI	c.401delA	p.K134Rfs*14	chr6:163899920	chr6:163899920	frameshift	13.50
P8-F	RNF43	c.1976delG	p.G659Vfs*41	chr17:56435161	chr17:56435161	frameshift	33.66
P8-F	SETD2	c.4187delA	p.N1396Mfs*16	chr3:47161939	chr3:47161939	frameshift	6.50
P8-F	BLM	c.1544delA	p.N515Mfs*16	chr15:91304139	chr15:91304139	frameshift	11.92
P8-F	MAP2K2	c.806delC	p.P269Rfs*58	chr19:4099312	chr19:4099312	frameshift	19.73
P8-F	BAX	c.278delT	p.F93Sfs*40	chr19:49459495	chr19:49459495	frameshift	18.43
P8-F	ARID1A	c.827delG	p.G276Efs*87	chr1:27023716	chr1:27023716	frameshift	17.61
P8-F	KMT2B	c.6056delG	p.G2019Afs*21	chr19:36223502	chr19:36223502	frameshift	19.45
P8-F	PPARD	c.539A>C	p.K180T	chr6:35391837	chr6:35391837	missense	5.03
P8-F	ALK	c.3662T>C	p.L1221P	chr2:29436931	chr2:29436931	missense	15.30
P8-F	FANCE	c.401G>A	p.R134H	chr6:35423676	chr6:35423676	missense	18.75
P8-F	KMT2B	c.595C>T	p.R199W	chr19:36210844	chr19:36210844	missense	19.41
P8-F	EGFR	c.2402A>G	p.Y801C	chr7:55249104	chr7:55249104	missense	14.95
P8-F	PKHD1	c.3890C>T	p.A1297V	chr6:51890718	chr6:51890718	missense	15.24
P8-F	TSC1	c.1238A>G	p.Q413R	chr9:135785983	chr9:135785983	missense	16.74
P8-F	FGFR1	c.2347A>G	p.T783A	chr8:38271268	chr8:38271268	missense	16.07
P8-F	CYP2D6	c.191G>A	p.R64H	chr22:42525901	chr22:42525901	missense	17.25
P8-F	LZTR1	c.278A>G	p.N93S	chr22:21340144	chr22:21340144	missense	18.04
P8-F	FBXW7	c.1429G>A	p.G477S	chr4:153247373	chr4:153247373	missense	13.10
P8-F	FANCE	c.163T>C	p.W55R	chr6:35420485	chr6:35420485	missense	12.81
P8-F	GRIN2A	c.3169G>A	p.A1057T	chr16:9858232	chr16:9858232	missense	16.47
P8-F	KDR	c.1991G>A	p.R664H	chr4:55968672	chr4:55968672	missense	12.94
P8-F	BAI3	c.622G>A	p.D208N	chr6:69349189	chr6:69349189	missense	14.53
P8-F	EPHA3	c.2932A>G	p.N978D	chr3:89528632	chr3:89528632	missense	15.54
P8-F	PTEN	c.485A>G	p.D162G	chr10:89693001	chr10:89693001	missense	17.01
P8-F	ERBB4	c.1178G>A	p.R393Q	chr2:212570063	chr2:212570063	missense	14.29
P8-F	MITF	c.938G>A	p.R313Q	chr3:70014077	chr3:70014077	missense	16.30

P8-F	ARID1A	c.4475C>T	p.A1492V	chr1:27101193	chr1:27101193	missense	17.07
P8-F	CTCF	c.883A>G	p.K295E	chr16:67645955	chr16:67645955	missense	16.80
P8-F	FANCA	c.1967C>T	p.A656V	chr16:89839726	chr16:89839726	missense	7.46
P8-F	FGFR2	c.181C>T	p.R61C	chr10:123325147	chr10:123325147	missense	2.17
P8-F	CSF1R	c.274G>A	p.G92R	chr5:149460363	chr5:149460363	missense	15.99
P8-F	SMAD4	c.260G>A	p.R87Q	chr18:48575066	chr18:48575066	missense	16.22
P8-F	ATM	c.8786G>A	p.R2929K	chr11:108224607	chr11:108224607	missense	8.57
P8-F	PAX5	c.691C>T	p.Q231*	chr9:36966635	chr9:36966635	nonsense	15.83
P8-F	CASP8	c.1294C>T	p.R432*	chr2:202150030	chr2:202150030	nonsense	18.40
P9-F	FLCN	c.1285delC	p.H429Tfs*39	chr17:17119709	chr17:17119709	frameshift	28.01
P9-F	MLH3	c.1755delA	p.E586Nfs*24	chr14:75514604	chr14:75514604	frameshift	25.82
P9-F	MSH6	c.3261delC	p.F1088Sfs*2	chr2:48030640	chr2:48030640	frameshift	27.91
P9-F	CASP8	c.1087delG	p.D363Ifs*19	chr2:202149819	chr2:202149819	frameshift	28.06
P9-F	KDM5A	c.3597delA	p.G1200Dfs*9	chr12:416953	chr12:416953	frameshift	21.26
P9-F	AXL	c.874delC	p.H292Ifs*5	chr19:41743933	chr19:41743933	frameshift	26.47
P9-F	BAX	c.121delG	p.E41Rfs*19	chr19:49458971	chr19:49458971	frameshift	27.36
P9-F	CHD4	c.218delA	p.K73Rfs*129	chr12:6711546	chr12:6711546	frameshift	21.71
P9-F	BRAF	c.1208dupC	p.A404Cfs*9	chr7:140482926	chr7:140482927	frameshift	22.65
P9-F	AMER1	c.519delT	p.F173Lfs*36	chrX:63412648	chrX:63412648	frameshift	49.39
P9-F	ARID2	c.5333delA	p.N1778Ifs*13	chr12:46287469	chr12:46287469	frameshift	25.90
P9-F	ETV4	c.399dupC	p.R134Qfs*49	chr17:41610700	chr17:41610701	frameshift	25.29
P9-F	PLK1	c.1051delC	p.L351Cfs*104	chr16:23698800	chr16:23698800	frameshift	28.83
P9-F	HNF1A	c.872delC	p.P291Qfs*51	chr12:121432118	chr12:121432118	frameshift	25.82
P9-F	IDH1	c.96delT	p.Y34Tfs*11	chr2:209116180	chr2:209116180	frameshift	25.76
P9-F	ARID1A	c.1848delC	p.S617Qfs*2	chr1:27059207	chr1:27059207	frameshift	25.36
P9-F	ETV6	c.885_904delCGGGCTGCATAGGGAAGGGA	p.D295Efs*25	chr12:12022776	chr12:12022795	frameshift	20.51
P9-F	RNF43	c.1976delG	p.G659Vfs*41	chr17:56435161	chr17:56435161	frameshift	53.67
P9-F	WISP3	c.678dupA	p.C227Mfs*21	chr6:112389433	chr6:112389434	frameshift	24.03
P9-F	TTF1	c.668delA	p.K223Rfs*45	chr9:135277541	chr9:135277541	frameshift	26.66
P9-F	ARID1A	c.3977delC	p.P1326Rfs*155	chr1:27100176	chr1:27100176	frameshift	25.39
P9-F	AXIN2	c.1419_1421dupCCA	p.H474dup	chr17:63533732	chr17:63533733	inframe insertion	18.44
P9-F	WRN	c.1530_1532dupAGA	p.E510dup	chr8:30945376	chr8:30945377	inframe insertion	17.69
P9-F	AMER1	c.2768C>A	p.A923D	chrX:63410399	chrX:63410399	missense	57.42
P9-F	CUL3	c.1520T>C	p.V507A	chr2:225365170	chr2:225365170	missense	32.57
P9-F	MGMT	c.344T>C	p.L115P	chr10:131506284	chr10:131506284	missense	29.48
P9-F	FBXW7	c.1429G>A	p.G477S	chr4:153247373	chr4:153247373	missense	25.28
P9-F	SMO	c.1320G>T	p.K440N	chr7:128848655	chr7:128848655	missense	5.76
P9-F	PARK2	c.362T>C	p.V121A	chr6:162683607	chr6:162683607	missense	25.99
P9-F	NSD1	c.4728G>A	p.M1576I	chr5:176678817	chr5:176678817	missense	28.43
P9-F	MAP3K4	c.512G>A	p.G171D	chr6:161469816	chr6:161469816	missense	21.89
P9-F	EPHA5	c.2540C>A	p.P847Q	chr4:66213890	chr4:66213890	missense	27.78
P9-F	GNAS	c.739G>A	p.A247T	chr20:57429059	chr20:57429059	missense	4.94
P9-F	HNF1A	c.1719_1720invCA	p.S574G	chr12:121437381	chr12:121437382	missense	28.84
P9-F	PIK3CA	c.2504C>A	p.P835H	chr3:178947068	chr3:178947068	missense	31.21
P9-F	KMT2C	c.878C>T	p.A293V	chr7:151970924	chr7:151970924	missense	5.76
P9-F	FGFR1	c.1409G>A	p.R470H	chr8:38275767	chr8:38275767	missense	22.55
P9-F	TERT	c.2105C>T	p.P702L	chr5:1279431	chr5:1279431	missense	27.78
P9-F	MAP2K2	c.360G>T	p.Q120H	chr19:4110597	chr19:4110597	missense	27.07
P9-F	GNAQ	c.1055A>C	p.N352T	chr9:80336264	chr9:80336264	missense	29.99
P9-F	TSC2	c.5131G>A	p.V1711M	chr16:2138111	chr16:2138111	missense	6.34

P9-F	SRC	c.422C>T	p.A141V	chr20:36022369	chr20:36022369	missense	27.12
P9-F	GNAS	c.305C>T	p.A102V	chr20:57478633	chr20:57478633	missense	28.61
P9-F	PDGFRB	c.2317G>A	p.A773T	chr5:149501470	chr5:149501470	missense	26.27
P9-F	LRP1B	c.6509G>C	p.C2170S	chr2:141458109	chr2:141458109	missense	30.01
P9-F	TSHR	c.1912G>A	p.A638T	chr14:81610314	chr14:81610314	missense	26.19
P9-F	POLD1	c.644C>T	p.A215V	chr19:50905516	chr19:50905516	missense	26.45
P9-F	KDM5A	c.1061T>C	p.F354S	chr12:461459	chr12:461459	missense	43.39
P9-F	CTNNB1	c.1457G>A	p.R486H	chr3:41275291	chr3:41275291	missense	27.66
P9-F	KDM5A	c.3092G>A	p.R1031H	chr12:420175	chr12:420175	missense	4.49
P9-F	B2M	c.68-2A>G	-	chr15:45007619	chr15:45007619	splice acceptor	27.47
P9-F	BLM	c.1423G>T	p.G475*	chr15:91304026	chr15:91304026	nonsense	28.55
P9-F	ARID2	c.5260C>T	p.R1754*	chr12:46287315	chr12:46287315	nonsense	31.40
P9-F	ARID1B	c.5791C>T	p.R1931*	chr6:157528105	chr6:157528105	nonsense	21.37
P9-F	BRCA1	c.3679C>T	p.Q1227*	chr17:41243869	chr17:41243869	nonsense	27.91

Note:

F: formalin-fixed paraffin-embedded; T: tissue; PLA: plasma.
